# Exploring the shared pathogenic mechanisms of tuberculosis and COVID-19: emphasizing the role of VNN1 in severe COVID-19

**DOI:** 10.3389/fcimb.2024.1453466

**Published:** 2024-11-21

**Authors:** Peng Sun, Yue Wang, Sijing Zhou, Jiahui Liang, Binbin Zhang, Pulin Li, Rui Han, Guanghe Fei, Chao Cao, Ran Wang

**Affiliations:** ^1^ Department of Respiratory and Critical Care Medicine, The First Affiliated Hospital of Anhui Medical University, Hefei, China; ^2^ Department of Infectious Diseases, Hefei Second People’s Hospital, Hefei, China; ^3^ Department of Occupational Disease, Hefei Third Clinical College of Anhui Medical University, Hefei, China; ^4^ Department of Breast Surgery, The First Affiliated Hospital of Anhui Medical University, Hefei, China; ^5^ Department of Respiratory and Critical Care Medicine, Key Laboratory of Respiratory Disease of Ningbo, The First Affiliated Hospital of Ningbo University, Ningbo, Zhejiang, China

**Keywords:** COVID-19, tuberculosis, machine learning, single-cell sequencing, VNN1, molecular docking, immune infiltration, mechanical ventilation

## Abstract

**Background:**

In recent years, COVID-19 and tuberculosis have emerged as major infectious diseases, significantly contributing to global mortality as respiratory illnesses. There is increasing evidence of a reciprocal influence between these diseases, exacerbating their incidence, severity, and mortality rates.

**Methods:**

This study involved retrieving COVID-19 and tuberculosis data from the GEO database and identifying common differentially expressed genes. Machine learning techniques, specifically random forest analysis, were applied to pinpoint key genes for diagnosing COVID-19. The Cibersort algorithm was employed to estimate immune cell infiltration in individuals with COVID-19. Additionally, single-cell sequencing was used to study the distribution of VNN1 within immune cells, and molecular docking provided insights into potential drugs targeting these critical prognosis genes.

**Results:**

GMNN, SCD, and FUT7 were identified as robust diagnostic markers for COVID-19 across training and validation datasets. Importantly, VNN1 was associated with the progression of severe COVID-19, showing a strong correlation with clinical indicators and immune cell infiltration. Single-cell sequencing demonstrated a predominant distribution of VNN1 in neutrophils, and molecular docking highlighted potential pharmacological targets for VNN1.

**Conclusions:**

This study enhances our understanding of the shared pathogenic mechanisms underlying tuberculosis and COVID-19, providing essential insights that could improve the diagnosis and treatment of severe COVID-19 cases.

## 1 Introduction

The 2019 coronavirus disease (COVID-19), caused by the severe acute respiratory syndrome coronavirus 2 (SARS-CoV-2), is an acute respiratory infectious disease. As of July 2023, this virus has spread globally at an alarming rate, leading to over 500 million infections and 6 million deaths (D’Souza et al., 2023). According to the World Health Organization (WHO), common symptoms include fever, dry cough, sore throat, diarrhea, fatigue, and joint and muscle pain (Sohrabi et al., 2020; Hu et al., 2021). Severe cases often develop acute respiratory distress syndrome (ARDS) and respiratory failure, necessitating ICU admission and mechanical ventilation, though outcomes are frequently poor (Wang et al., 2020). The virus’s propensity for mutation and rapid transmission, coupled with challenges in vaccine development, suggests that COVID-19 may persist among human populations for an extended period, posing significant challenges to controlling other diseases.

Tuberculosis (TB), a longstanding respiratory disease, continues to affect millions, with over ten million new cases reported annually (Furin et al., 2019). Since the onset of the COVID-19 pandemic, TB diagnosis and treatment have been affected, as evidenced by data from 16 countries (Migliori et al., 2020). Projections suggest a potential 13% increase in TB-related deaths in the coming year (Chakaya et al., 2021). Increasingly, evidence highlights a correlation between COVID-19 and TB. Reports from the WHO and studies from regions with high TB burdens like Italy and China indicate that TB patients are at increased risk of contracting COVID-19 (Harding, 2020; Stochino et al., 2020; Shah et al., 2022). This co-infection increases mortality, with TB raising the death rate among COVID-19 patients by significant margins (Gao et al., 2021; Risk factors for coronavirus disease 2019 (COVID-19) death in a population cohort study from the western cape province, South Africa, 2021).

**Figure 1:**
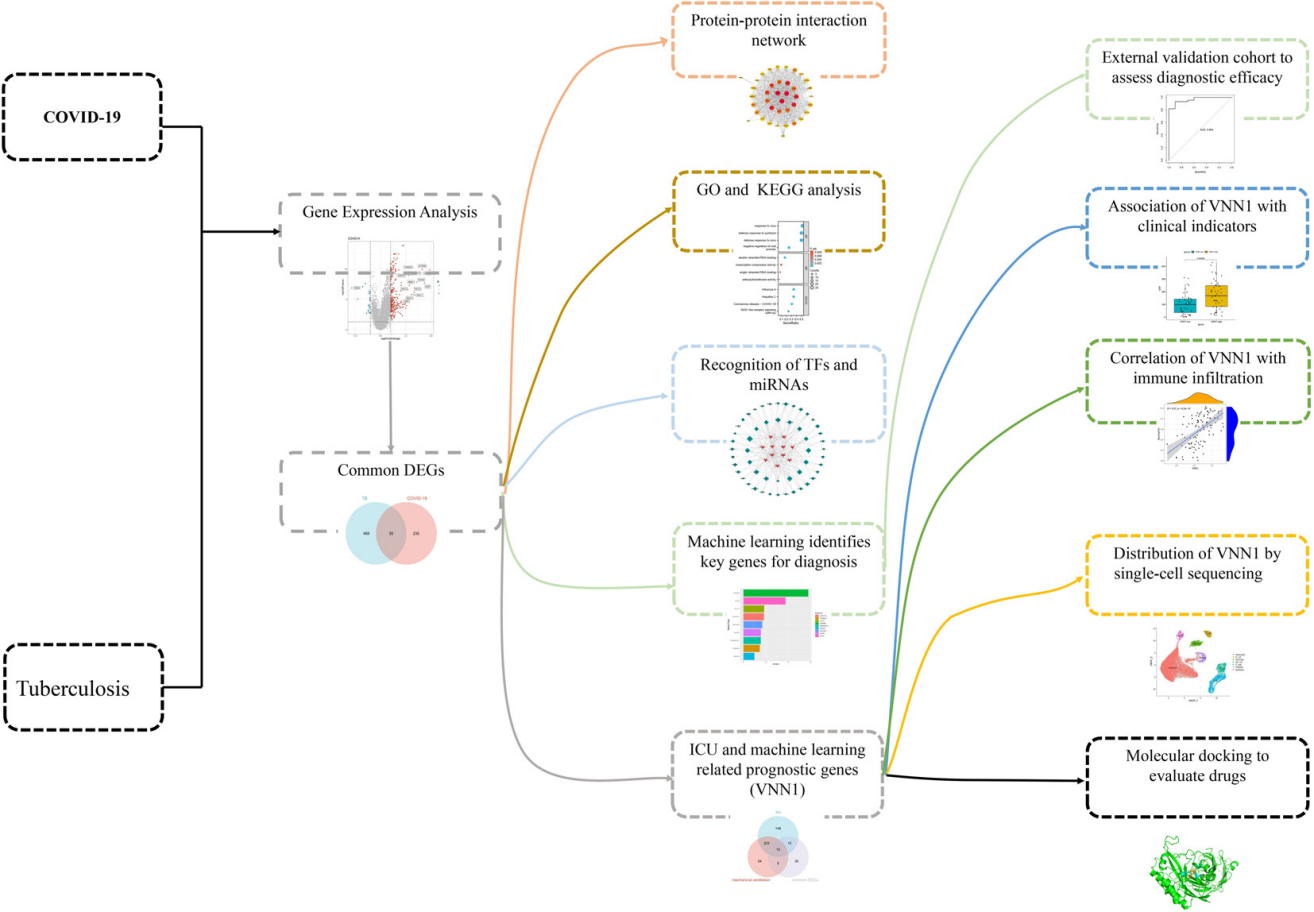
Schematic illustration of the overall general workflow of this study.

This research aims to elucidate the shared pathogenic mechanisms between COVID-19 and TB by analyzing 55 commonly differentially expressed genes. We conducted protein-protein interaction (PPI) network analysis and Gene Ontology (GO)/Kyoto Encyclopedia of Genes and Genomes (KEGG) enrichment analysis on these genes. We also explored transcriptional regulation by investigating transcription factors and miRNAs likely involved. Diagnostic genes for COVID-19 were identified using machine learning techniques, with validation achieved in a separate dataset. Additionally, we analyzed genes related to severe COVID-19 outcomes, such as ICU admission and mechanical ventilation requirements. Our findings reveal a significant association between the gene VNN1 and key clinical and prognostic indicators. Single-cell sequencing showed VNN1 predominantly expressed in neutrophils, potentially influencing other immune cells and affecting COVID-19 prognosis. Through molecular docking, we identified potential drugs targeting VNN1, aiming for their future clinical use. The sequence of our research activities is detailed in **Figure 1**.

## 2 Materials and methods

### 2.1 Data acquisition

The Gene Expression Omnibus (GEO) database, a comprehensive resource for gene expression data, was used to acquire the datasets for this study. The tuberculosis data was obtained from GSE19491, while the COVID-19 data were derived from five datasets: GSE157103, GSE171110, GSE217948, GSE164805, and GSE152418. Detailed information can be found in the **Supplementary Data Sheet 1**. Differentially expressed genes (DEGs) for tuberculosis were selected with an adjusted P-value (adj.P) less than 0.05 and a log2 fold change (log2FC) of at least 0.5. For COVID-19, DEGs were identified using the same adj.P criterion and a log2FC of at least 1.

### 2.2 Functional enrichment analysis of common DEGs

The ‘clusterProfiler’ package in RStudio (version 4.2.2) facilitated the exploration of biological processes associated with COVID-19 and TB common DEGs through Gene Ontology (GO) and Kyoto Encyclopedia of Genes and Genomes (KEGG) enrichment analyses.

### 2.3 Protein-protein interaction analysis

The String database was employed to analyze interactions among proteins encoded by common DEGs shared between COVID-19 and tuberculosis. Visualizations were conducted using Cytoscape (version 3.8.4).

### 2.4 Transcription factors and miRNAs

Networks involving DEGs and transcription factors (TFs), and miRNAs were constructed using the NetworkAnalyst platform, with TFs identified through the JASPAR database (Mahmud et al., 2021) and miRNA-DEG relationships sourced from the miRTarBase database (Hsu et al., 2011). Visualizations were performed using Cytoscape.

### 2.5 Identification of key diagnostic genes using machine learning

The random forest (RF) machine learning method, a robust approach involving the construction of multiple decision trees, was utilized to identify key genes for COVID-19 diagnosis. The ‘classif.ranger’ learner from the mlr3 R package was used to score each DEG. Diagnostic performance was evaluated using ROC curves constructed with the ‘pROC’ R package.

### 2.6 Identification of key prognostic genes associated with clinical information

Clinical data were collected from dataset GSE157103, which included various metrics such as C-reactive protein, D-dimer, and ICU admission status. Prognostic groups were defined based on ICU admission and mechanical ventilation use. Key prognostic genes were identified at the intersection of DEGs between these groups, with an adj.P less than 0.05 and a log2FC of at least 1.

### 2.7 Analysis of VNN1 and immune cells

The CIBERSORT tool was used to estimate the composition of immune cells and analyze gene expression profiles. The correlation between VNN1 expression and immune cells was assessed using the Spearman correlation method.

### 2.8 Acquisition and processing of single-cell data

Single-cell RNA sequencing data were obtained from the GEO database under accession number GSE157344. This dataset includes 20 samples from critically ill COVID-19 patients and 6 peripheral blood samples from healthy controls. Initial data processing involved filtering based on cell count, feature count, and the proportion of mitochondrial genes. Data were then normalized using the NormalizeData function. Dimensionality reduction was performed using the RunPCA function, which focused on 2000 highly variable genes and the top 10 principal components. Visualization was achieved through UMAP using the RunUMAP function, and cell types were annotated using the SingleR package.

### 2.9 Evaluation of applicant drugs and molecular docking

Potential functional molecules associated with VNN1 were explored using the Enrichr portal and the DSigDB. Molecular docking, a key computational method in drug discovery, was employed to predict interactions between ligands and the VNN1 target. The VNN1 crystal structure was downloaded from the Protein Data Bank (PDB code 7sly) to guide the docking analysis. Drug structures targeting VNN1 were sourced from the PubChem database. AutoDock software was used for docking, assessing binding energy reliability and ligand positioning accuracy. Docking results were visualized using PyMOL software.

### 2.10 qRT-qPCR

Sample collection: Peripheral blood samples were obtained from the First Affiliated Hospital of Anhui Medical University, including 6 healthy controls, 10 mild COVID-19 patients, and 6 severe COVID-19 patients. Severe COVID-19 was defined by meeting any of the following criteria: 1) SpO2 < 94% on room air at sea level; 2) PaO2/FiO2 < 300 mm Hg; 3) respiratory frequency > 30 breaths/min; 4) lung infiltrates > 50% (Attaway et al., 2021) and requiring mechanical ventilation. Patients not meeting these criteria were classified as mild COVID-19.

RNA extraction and cDNA synthesis: Total RNA was extracted from peripheral blood cells using TRIzol reagent (Biosharp, Hefei, China). cDNA was synthesized using Hifair^®^ II 1st Strand cDNA Synthesis SuperMix for qPCR (YEASEN) according to the manufacturer’s instructions. qRT-PCR: Quantitative PCR was performed on a Roche LightCycler 96 system (Roche, Basel, Switzerland) using Hieff^®^ qPCR SYBR Green Master Mix (No Rox) (YEASEN). The cycling conditions were: initial denaturation at 95°C for 5 min, followed by 40 cycles of 95°C for 10 sec and 60°C for 30 sec. Primer sequences for VNN1 were: Forward 5’-TCCTGAGGTGTTGCTGAGTG-3’; Reverse Primer: 5’-AGCGTCCGTCAGTTGACAC-3’.GAPDH was used as an internal control, with primer sequences: Forward 5’-AGGTCGGTGTGAACGGATTTG-3’; Reverse 5’-TGTAGACCATGTAGTTGAGGTCA-3’.

Data analysis: Relative gene transcription levels was calculated using the 2^-ΔΔCt method, where ΔCt = Ct(VNN1) - Ct(GAPDH), and ΔΔCt = ΔCt(COVID group) - ΔCt(control group). All experiments were performed in triplicate. Detailed primer information is provided in **Supplementary Data Sheet 2**.

### 2.11 Statistical analysis

Data processing and statistical analyses were conducted using R software version 4.2.2. The Wilcoxon rank-sum test was employed to compare two groups, while relationships between continuous variables were assessed using Spearman correlation. Random forest analysis was carried out using the mlr3 R package, and single-cell RNA sequencing data were analyzed with the Seurat package. All statistical tests were two-tailed, with a significance threshold set at P < 0.05.

## 3 Result

### 3.1 Identification and functional enrichment analysis of COVID-19 and TB common DEGs

**Figure 2:**
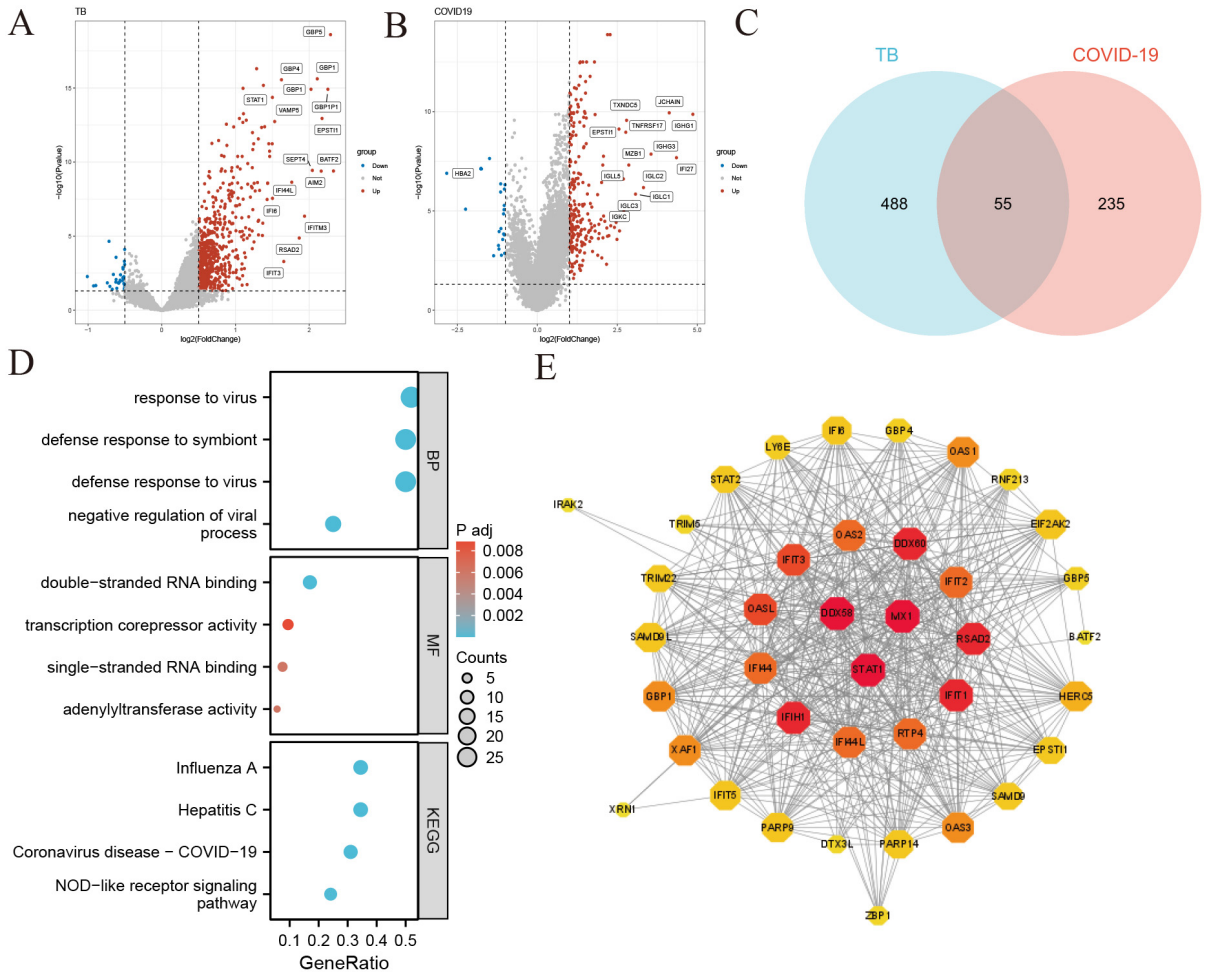
Identification of common differentially expressed genes (DEGs) and protein-protein interaction (PPI) network in COVID-19 and tuberculosis **(A)** Volcano plot illustrating the distribution of DEGs in tuberculosis. Red dots represent significantly upregulated genes, blue dots represent significantly downregulated genes, and gray dots indicate genes without significant change. **(B)** Volcano plot depicting DEGs in COVID-19 **(C)** Venn diagram showing the overlap of DEGs between COVID-19 and tuberculosis. **(D)** Bubble graphs presenting the top enriched Gene Ontology (GO) terms and Kyoto Encyclopedia of Genes and Genomes (KEGG) pathways. The size of each bubble represents the number of genes involved, while the color indicates the significance level. **(E)** Protein-Protein Interaction (PPI) network of the common DEGs between COVID-19 and tuberculosis. Node size and color depth indicate the degree of protein interaction, with larger and darker nodes representing more significant interactions.

To explore the relationship between tuberculosis (TB) and COVID-19, we analyzed blood sample data from both diseases sourced from the GEO database. We identified 680 DEGs in the TB dataset and 290 in the COVID-19 dataset. The overall transcriptional gene expression profiles for both diseases are visually represented in **Figures 2A, B**. A Venn diagram in **Figure 2C** shows 55 DEGs common to both diseases (**Supplementary Data Sheet 3**). Functional enrichment analyses were conducted using KEGG pathways and GO terms (**Supplementary Data Sheet 4**). The top five significant pathways are detailed in **Figure 2D**. GO analysis focused on biological processes, revealing that these DEGs are primarily involved in the organism’s response to viruses, encompassing cellular and molecular reactions to viral infections. The molecular functions of these genes are linked to RNA transcription and binding, impacting gene regulation, RNA synthesis, and interactions. Notably, the NOD-like receptor signaling pathway, critical for immune and inflammatory responses, was identified as a key pathway activated by these DEGs.

### 3.2 Protein-protein interaction network analysis

We constructed a Protein-Protein Interaction (PPI) network using the common DEGs between COVID-19 and TB, depicted in **Figure 2E**. The network visualization utilizes circle size and color depth to indicate the degree of protein interaction; larger and darker circles signify more significant interactions. Key proteins include DDX58, STAT1, MX1, IFIH1, IFIT1, RSAD2, DDX60, OASL, IFIT3, and RTP4, recognized for their central roles and strong associations within the network.

### 3.3 Construction of regulatory networks

**Figure 3:**
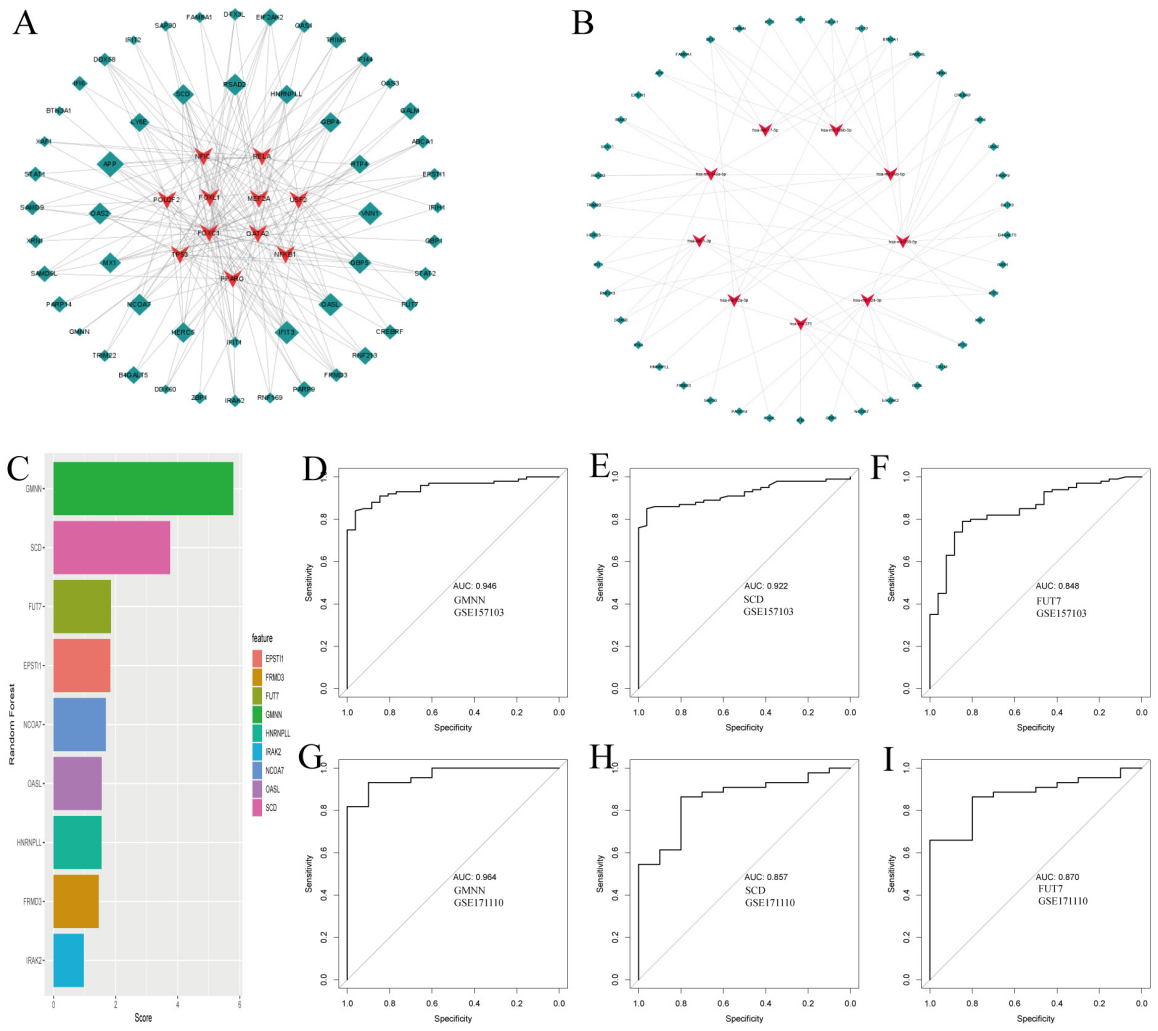
Regulatory gene networks of common differentially expressed genes and diagnostic efficacy of selected biomarkers in COVID19. **(A)** Interconnected regulatory interaction network for the common DEGs and their associated transcription factors (TFs). Transcription factors a are represented by red V-shaped nodes, while the DEGs are depicted as green diamonds, with larger diamonds indicating stronger associations. **(B)** Regulatory interaction network illustrating the relationships between the common DEGs and their corresponding miRNAs, using the same color scheme as panel **(A)**. **(C)** Feature selection using Random Forest, shown as a bar chart ranking the diagnostic efficacy of selected genes for COVID-19. **(D-F)** AUC scores for the selected genes in the training set GSE157103, demonstrating their diagnostic performance. **(G-I)** AUC scores for the selected genes in the independent testing set GSE171110, validating their diagnostic utility.

Using NetworkAnalyst, we predicted interactions between the common DEGs and transcription factors (TFs), identifying the top 10 TFs based on their degree of connection with DEGs, as shown in **Figure 3A** (**Supplementary Data Sheet 5**). These TFs, including NFIC, RELA, POU2F2, FOXL1, MEF2A, USF2, FOXC1, GATA2, TP53, NFKB1, and PPARG, are crucial for their significant roles in gene regulation. Additionally, **Figure 3B** illustrates the interactions between miRNAs and the common DEGs, where red “V” shapes represent DEGs, and green squares represent miRNAs. This analysis sheds light on potential post-transcriptional regulatory mechanisms and therapeutic targets (**Supplementary Data Sheet 6**).

### 3.4 Identifying hub genes for diagnosis based on COVID-19 and TB common DEGs

Following our analysis of DEGs common to COVID-19 and tuberculosis, we explored the potential impacts of these genes on COVID-19 disease processes through various pathways and mechanisms. Using the random forest method for feature selection, implemented via the mlr3 R package, we ranked genes based on their importance, focusing on those with a Gini index exceeding a predefined threshold of 1.0 (**Figure 3C**). We identified three top-ranked genes—GMNN, SCD, and FUT7—as crucial diagnostic markers. These genes demonstrated excellent diagnostic performance in the training set, with AUC values of 0.946, 0.922, and 0.848 respectively (**Figures 3D–F**). In the validation set, they continued to show promising results, with AUC values of 0.964, 0.857, and 0.870 respectively (**Figures 3G–I**). The robust performance of these genes underscores their potential key role in the pathogenesis of COVID-19 and provides valuable insights for further investigation into their specific mechanisms within the disease.

### 3.5 Identification and validation of genes associated with severe COVID-19

**Figure 4:**
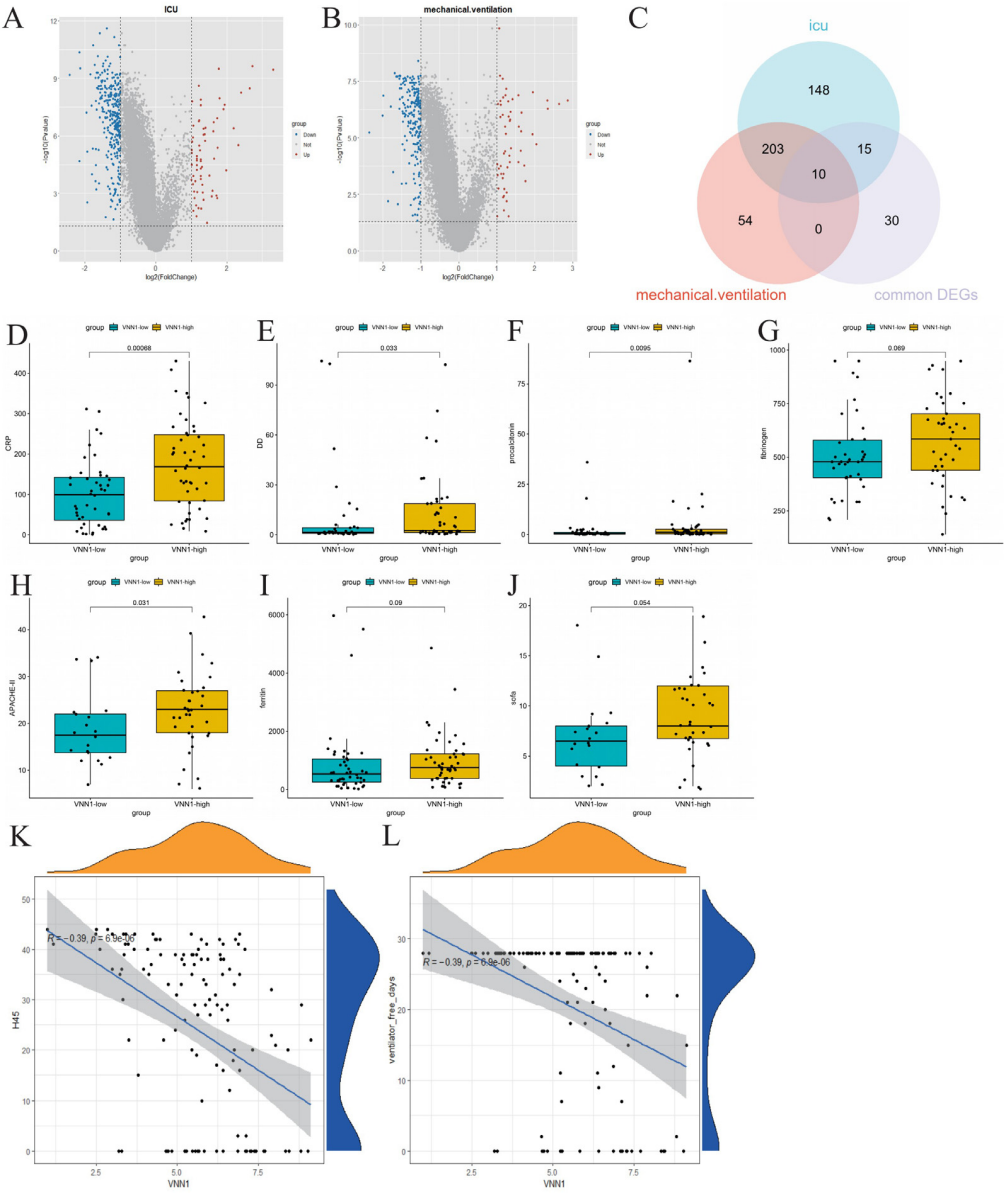
Identification of genes related to severe COVID-19 and their correlation with clinical indicators. **(A)** Volcano plot of differentially expressed genes in ICU patients, highlighting significant upregulated and downregulated genes. **(B)** Volcano plot of differentially expressed genes in mechanically ventilated patients. **(C)** Venn diagram showing the intersection of common differentially expressed genes, differentially expressed genes associated with ICU admission, and differentially expressed genes related to mechanical ventilation. **(D–J)** Comparison of clinical indicators (CRP, D-dimer, procalcitonin, fibrinogen, APACHE-II, ferritin, and SOFA scores) between high and low expression groups of VNN1. **(K, L)** Correlation analysis between VNN1 expression and the number of days without hospitalization or mechanical ventilation within 45 days post-admission.

We stratified COVID-19 patients based on ICU admission and the use of mechanical ventilation to identify molecular signatures associated with severe disease outcomes. Differential gene expression analysis revealed 376 genes differentially expressed between the ICU and non-ICU groups (**Figure 4A**), and 267 genes between the mechanically ventilated and non-ventilated groups (**Figure 4B**). A Venn diagram analysis helped us identify 10 crucial genes (VNN1, GBP4, XAF1, OAS1, OAS2, OAS3, RTP4, IFI44L, IFIT1 and RSAD2) intersecting with the commonly expressed DEGs in both COVID-19 and tuberculosis (**Figure 4C**).

Subsequently, we investigated the relationship between the identified genes and various clinical indicators, including CRP, D-dimer, ferritin, fibrinogen, APACHE II score, and SOFA score. Remarkably, only the transcript levels level of VNN1 demonstrated a significant correlation with these clinical indicators. (**Figures 4D–J**). We also explored the relationship between the transcript levels of VNN1 and specific clinical outcomes, including the number of hospital-free days within 45 days post-admission (H45) (**Figure 4K**) and the number of days off mechanical ventilation (DOMV) (**Figure 4L**). A negative correlation was observed between high VNN1 transcript levels and both H45 and DOMV, suggesting its significance in predicting poorer outcomes in severe COVID-19 cases.

**Figure 5:**
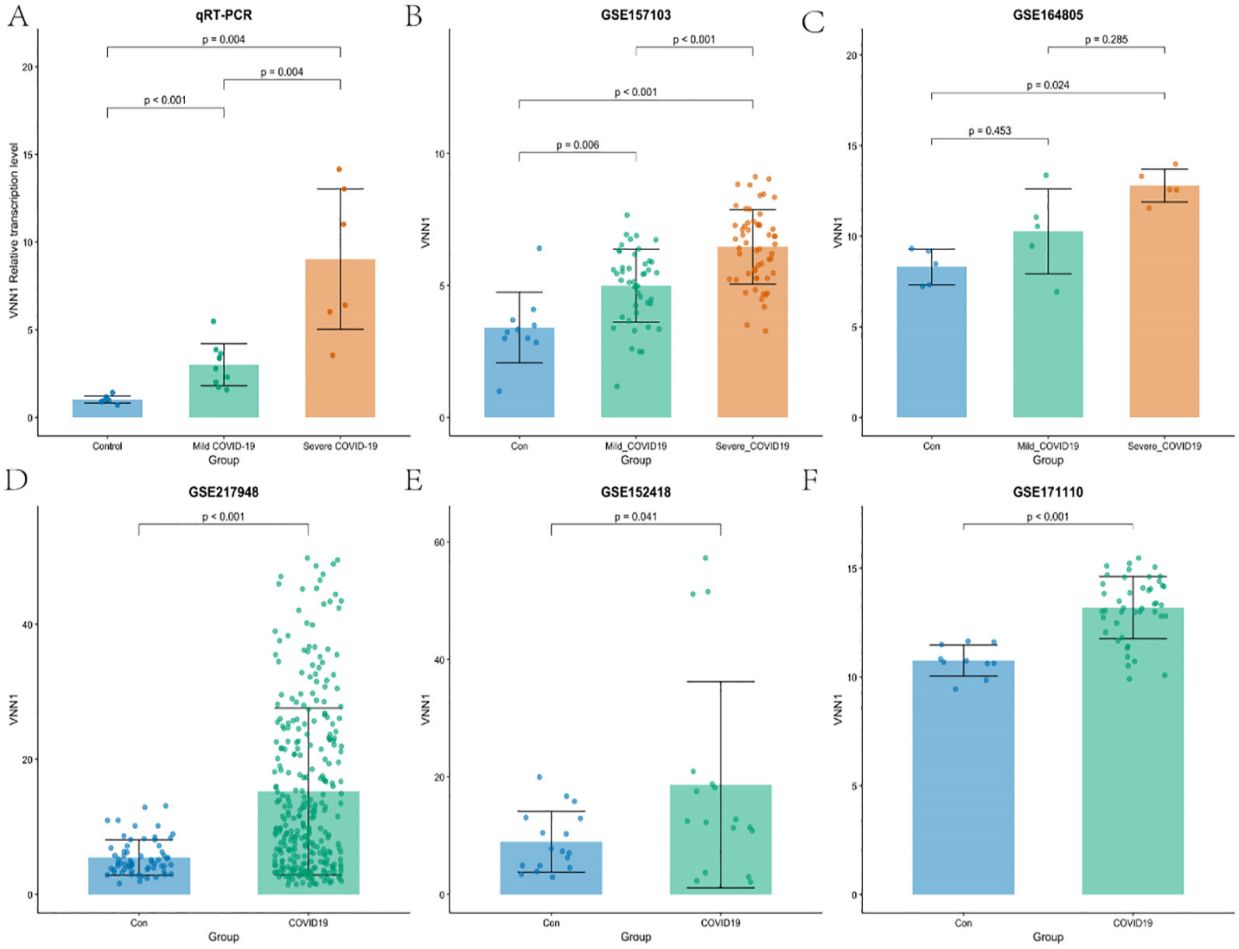
Validation of VNN1 transcription through qRT-PCR and additional datasets. **(A)** Validation of VNN1 transcription levels using qRT-PCR in a cohort from The First Affiliated Hospital of Anhui Medical University. **(B)** VNN1 transcription levels analysis in dataset GSE157103. **(C)** VNN1 transcription levels analysis in dataset GSE164805. **(D)** VNN1 transcription levels analysis in dataset GSE217948. **(E)** VNN1 transcription levels analysis in dataset GSE152418. **(F)** VNN1 transcription levels analysis in dataset GSE171110.

To further validate our results, we examined the transcription level of VNN1 across various datasets, including the cohort from the First Affiliated Hospital of Anhui Medical University. Our analysis revealed a significant increase in VNN1 transcription levels in COVID-19 patients compared to the control group. Notably, this elevation was particularly pronounced in patients with severe COVID-19, as illustrated in **Figures 5A–F**.

In the qRT-PCR analysis (**Figure 5A**), the relative transcription levels of VNN1 were significantly higher in the severe COVID-19 group (p < 0.001) compared to both the control and mild COVID-19 groups, indicating a potential correlation between VNN1 expression and disease severity.

Similarly, in the GSE157103 dataset (**Figure 5B**), VNN1 levels were significantly elevated in severe cases (p < 0.001), with a marked difference compared to mild cases (p = 0.006). In the GSE164805 dataset (**Figure 5C**), while there was an observable increase in VNN1 levels among mild COVID-19 patients, this did not reach statistical significance (p = 0.453). This lack of significance may be attributed to the small sample size, with only 5 samples in each group, which limits the statistical power to detect true differences. However, severe cases did show significantly higher transcript levels of VNN1 compared to the control group (p = 0.024). This highlights potential variability between different cohorts and reinforces the correlation between VNN1 expression and disease severity in severe patients. The GSE217948 dataset (**Figure 5D**) also demonstrated a significant upregulation of VNN1 in COVID-19 patients (p < 0.001), while the GSE152418 dataset (**Figure 5E**) showed a modest increase (p = 0.041). Finally, in the GSE171110 dataset (**Figure 5F**), VNN1 transcript levels was significantly higher in COVID-19 patients (p < 0.001), reinforcing our findings across multiple datasets.

These results collectively support the hypothesis that VNN1 transcription levels are associated with the severity of COVID-19, highlighting its potential role as a biomarker for disease progression.

### 3.6 The relationship between clinical information, immune infiltration, and VNN1 expression

**Figure 6:**
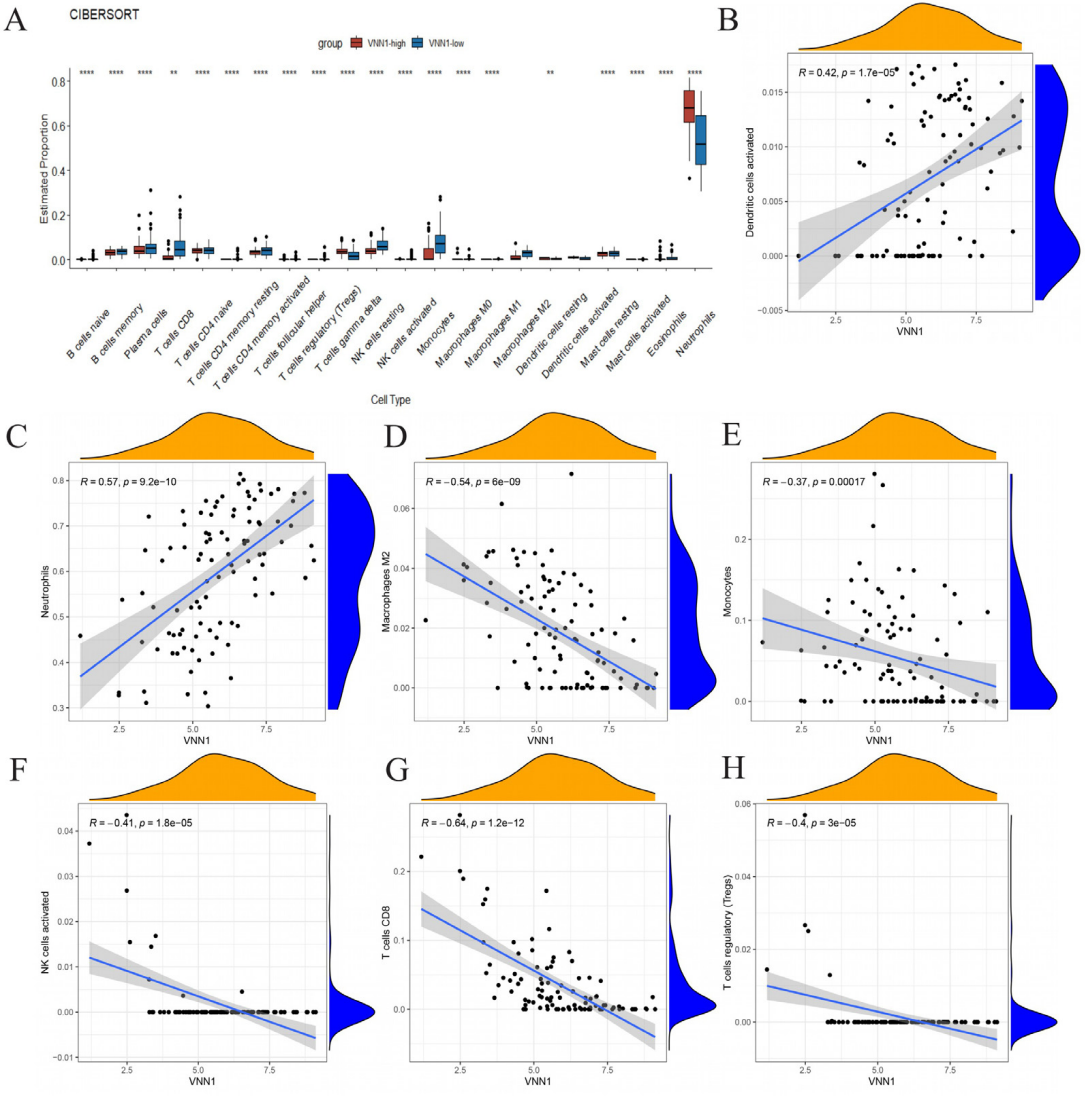
The association between VNN1 and immune cell infiltration. **(A)** Differences in immune cell infiltration within the VNN1 high and low expression groups. **(B–H)** The correlation between VNN1 expression and the infiltration of activated dendritic cells, neutrophils, M2 macrophages, monocytes, activated NK cells, CD8 T cells, and Treg cells. (“**” signifies a p-value < 0.01, “****” signifies a p-value < 0.0001).

We used the CIBERSORT algorithm to assess immune cell infiltration in COVID-19 patients (**Figure 6A**). Our correlation analysis showed a positive association between H45 and dendritic cells, NK cells, CD8 T cells, and Tregs cells, whereas M0 and M2 macrophages, as well as neutrophils, were negatively correlated with H45 (**Supplementary Data Sheet 7**). A similar trend was observed for DOMV, with positive correlations noted for resting NK cells and CD8 T cells and negative correlations for M0 macrophages and neutrophils (**Supplementary Data Sheet 8**). A strong positive correlation was identified between VNN1 expression and the presence of dendritic cells and neutrophils (**Figures 6B, C**), while a significant negative correlation was observed with monocytes, M2 macrophages, CD8 T cells, NK cells, and Treg cells (**Figures 6D–H**). These findings suggest that VNN1 could influence the prognosis of COVID-19 patients by modulating immune cell infiltration.

### 3.7 Expression and distribution of VNN1 in single cell sequencing

**Figure 7:**
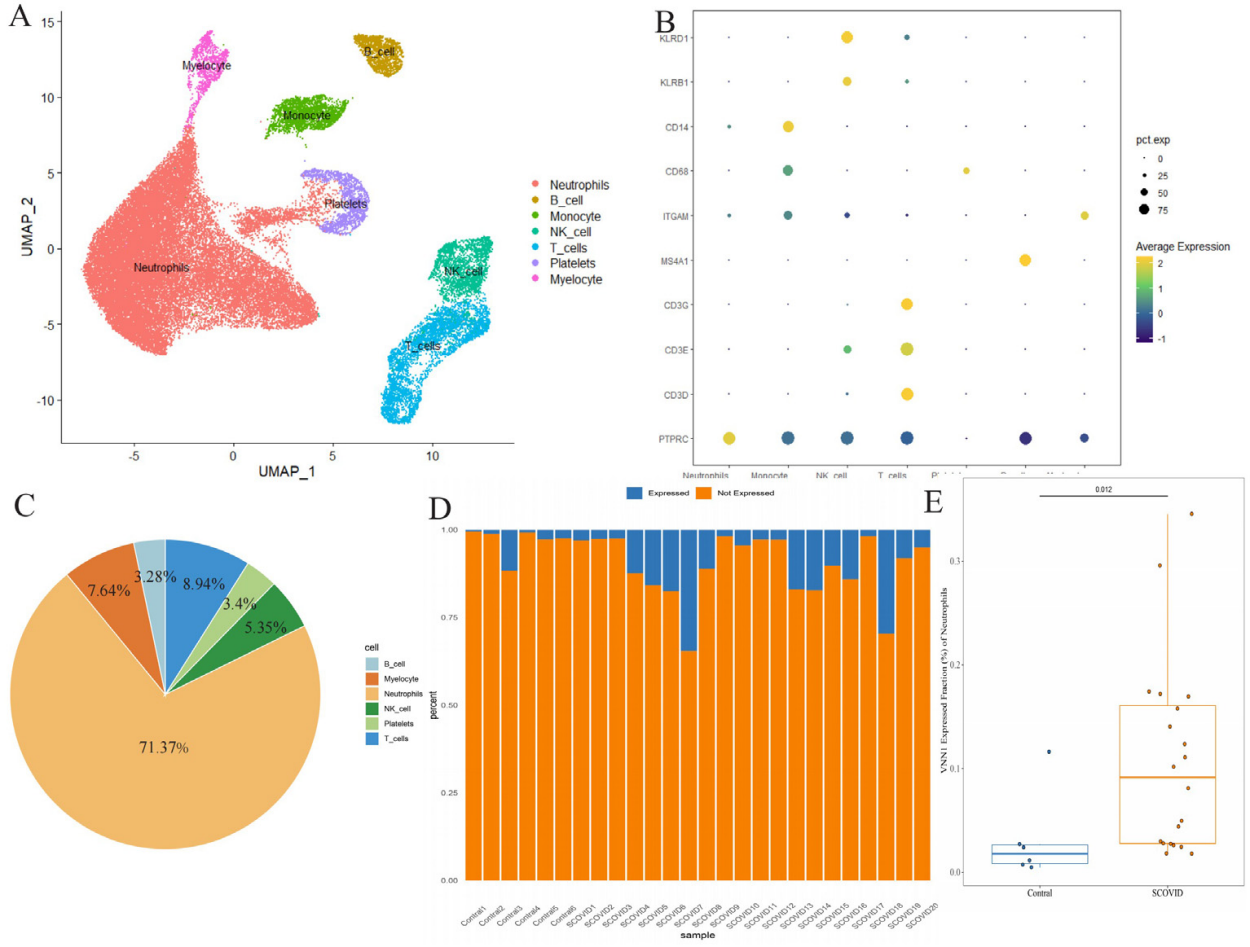
Differential expression of VNN1 in blood specimens **(A)** Cell type annotation of the single-cell data. **(B)** Expression of marker genes for different cell types, represented as a bubble chart where the color indicates expression levels and the size of the bubbles represents the percentage of cells expressing the marker. **(C)** Cell Type Distribution of VNN1-Expressing Cells **(D)** Proportion of neutrophils expressing VNN1 among different samples **(E)** The difference in the proportion of neutrophils expressing VNN1 between the Contral and Severe COVID19 groups.

Using single-cell sequencing, we annotated 44,405 cells from 20 severely ill COVID-19 patients and 6 healthy controls, categorizing them into distinct cell types like neutrophils, monocytes, B cells, myelocytes, platelets, T cells, and NK cells (**Figure 7A**). Marker genes for each cell subtype are presented in **Figure 7B**. Notably, VNN1 showed predominant expression in neutrophils (**Figure 7C**), significantly upregulated in severe COVID-19 cases compared to controls (**Figures 7D, E**).

### 3.8 Identification of candidate drugs and target–chemical interaction in COVID-19

**Figure 8:**
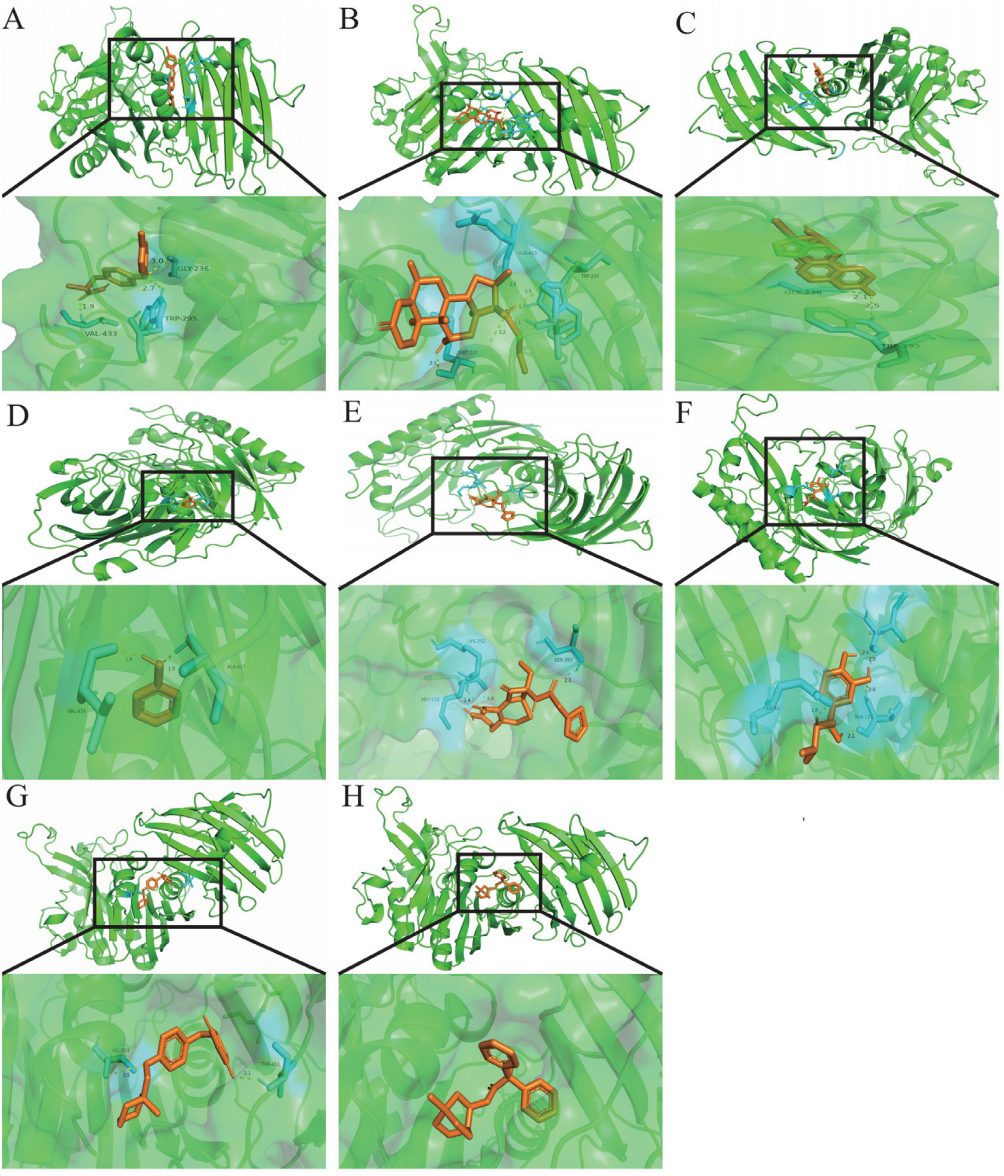
Molecular docking patterns.Molecular docking patterns for **(A)** Clofop, **(B)** fluticasone, **(C)** etynodiol, **(D)** phenol, **(E)** cefoxitin, **(F)** -isoprenaline, **(G)** ciglitazone, and **(H)** clidinium with the VNN1, respectively.

Exploring target–chemical interactions is crucial for clinical translation and the development of therapeutic drugs. We identified candidate drugs using Enrichr, focusing on compounds with the potential to impact severe COVID-19. The top ten drugs, including chloroform, Clofop, fluticasone, etynodiol, phenol, cefoxitin, (-)-isoprenaline, ciglitazone, tolazoline, and clidinium bromide were selected based on their p-values (**Supplementary Data Sheet 9**). Molecular docking predicted the binding modes between these compounds and the VNN1 protein, setting a binding energy criterion of ≤−5.0 kcal/mol to indicate potential efficacy. Clofop, fluticasone, etynodiol, phenol, cefoxitin, (-)-isoprenaline, ciglitazone, and clidinium bromide demonstrated promising interactions (**Table 1**), particularly etynodiol, which exhibited the lowest binding energy, indicating significant potential for further investigation (**Figures 8A–H**).

**Table 1:** The binding affinity of VNN1 with the docking compound.

compound	binding free energy
Choloroform	-3.38
Clofop	-5.5
fluticasone	-8.2
etynodiol	-11.32
phenol	-5.11
cefoxitin	-5.29
(-)-isoprenaline	-5.95
ciglitazone	-7.45
tolazoline	-4.63
clidinium bromide	-7.06

## 4 Discussion

The interaction between TB and COVID-19, especially their co-infection dynamics, has attracted increasing research interest due to the elevated incidence and mortality rates observed among patients suffering from both conditions (Hogan et al., 2020; Sy et al., 2020; Chakaya et al., 2021). Studies indicate that severe COVID-19 patients with concurrent TB are at a higher risk of mortality and experience a faster progression to death compared to those without TB (Sy et al., 2020; Gao et al., 2021). Our research aimed to explore the connections between COVID-19 and TB, uncovering shared pathogenic mechanisms and establishing a theoretical framework for diagnosis and treatment.

Our enrichment analysis of common differentially expressed genes revealed key pathways involved in both diseases, including viral response, RNA binding, and the NOD-like receptor signaling pathway. The innate immune system, which first responds to viral invasions, utilizes pathogen-recognition receptors (PRRs) such as C-type lectin receptors (CLRs), NOD-like receptors (NLRs), RIG-I-like receptors (RLRs), and Toll-like receptors (TLRs) to detect pathogen-associated molecular patterns (PAMPs) (Ishii et al., 2008; Tartey and Takeuchi, 2017). Subsequently, the adaptive immune response is activated, involving interactions between viral particles and antigen-presenting cells or B-cell receptors, which play crucial roles in the body’s defense against viral invasion (Primorac et al., 2022). Similarly, Mycobacterium tuberculosis exploits PRRs, including TLRs, NLRs, and CLRs, to invade and replicate within type II lung epithelial cells and alveolar macrophages (Liu et al., 2017).

The Protein-Protein Interaction analysis identified DDX58, MX1, and STAT1 as central genes in the context of COVID-19 and TB. DDX58 (RIG-I) plays a crucial role in the innate immune response against viral infections (Radzikowska et al., 2023), while MX1 is involved in cellular antiviral responses (Andolfo et al., 2021). STAT1 is essential for signaling from type I, II, and III interferons, impacting immune responses to both diseases (Tolomeo et al., 2022).

In our analysis of diagnostic markers, GMNN, SCD, and FUT7 emerged as promising candidates, demonstrating excellent performance in distinguishing COVID-19 cases. Given the importance of early identification in critical conditions, these markers may facilitate timely intervention and improve patient outcomes. Early identification and intervention in patients’ conditions become increasingly important in reducing the number of patients requiring ICU admission and mechanical ventilation. According to existing guidelines, some clinical indicators such as CRP, D-dimer, ferritin, neutrophils, and chemokines are associated with the severity of COVID-19 and patient mortality rates (Chen et al., 2020; Merad and Martin, 2020). In our study, we intersect the common differentially expressed genes of COVID-19 and tuberculosis with the differentially expressed genes associated with ICU admission and the use of mechanical ventilation. Subsequently, we will validate the relationship between the intersected genes and clinical indicators. Surprisingly, VNN1 is associated with most clinical indicators, including C-reactive protein (CRP), D-dimer, procalcitonin, and APACHE-II. Furthermore, we observed a significant negative correlation between VNN1 and the H45, as well as the DOMV. This suggests that VNN1 plays a crucial role in the progression of COVID-19 patients to a critical condition, indicating its potential as a biological marker for disease detection and as an important therapeutic target.

COVID-19, like tuberculosis, is marked by significant alterations in immune cell profiles, particularly a notable reduction in lymphocytes which are key indicators of the disease’s severity (Zhao et al., 2020). Our study observed that in patients with high VNN1 expression, there was a substantial decrease in B cells, CD4 T cells, CD8 T cells, NK cells, and M2 macrophages. Conversely, the proportion of neutrophils significantly increased, suggesting a shift towards a neutrophil-dominated immune response. At the cellular level, the body’s initial defense against viral entry is mediated by type I interferon (INF), primarily produced by dendritic cells (Cervantes-Barragan et al., 2007; Hosseini et al., 2020). This response activates the Janus kinase (JAK) and signal transducer and activator of transcription (STAT) pathways via the IFNα/β receptor (IFNAR), stimulating the production of IFN-induced transmembrane (IFITM) proteins that hinder viral replication (Huang et al., 2011; de Wit et al., 2016; Kindler et al., 2016). Moreover, macrophages infected by SARS-CoV-2 release a variety of cytokines and chemokines, including TNF-α, IL-6, IL-12, IFN-γ, CCL2, CCL5, and CXCL10 (Zhou et al., 2014). These cytokines not only help in fighting the virus but also promote the migration of immune cells like neutrophils and macrophages to the lungs, contributing to alveolar damage and vascular disruption, which can be fatal (Li et al., 2020).T cells, both CD4+ and CD8+, are crucial in the antiviral immune response, the former inducing T-dependent B cell responses and the latter eliminating virally infected cells. However, the presence of certain cytokines such as TNF-α, IL-6, and IL-10 and specific immune cells like Th17 can induce T cell necrosis or apoptosis, leading to a reduction in their numbers (Diao et al., 2020; Xu et al., 2020). This depletion not only facilitates viral persistence but also heightens the risk of opportunistic infections, such as tuberculosis, further complicating the patient’s condition.

Vanin-1 (VNN1), known for its role in stress and inflammatory responses through its pantetheinase activity, hydrolyzes pantetheine to produce cysteamine. While its functions have been explored in cancer (Kang et al., 2016) and pancreatic inflammation (Millet et al., 2023), its implications in pulmonary diseases remain under-explored. Our findings highlight VNN1’s association with key clinical markers and its correlation with increased neutrophils and decreased lymphocytes, indicating its critical role in exacerbating COVID-19.

To explore VNN1’s potential as a therapeutic target, we conducted compound screening and molecular docking analyses. Compounds such as clofop, fluticasone, etynodiol, phenol, cefoxitin, (-)-isoprenaline, ciglitazone, and clidinium bromide emerged as promising candidates that may influence VNN1 activity, opening avenues for novel therapeutic strategies against COVID-19.

Despite the significant findings of our study, we acknowledge several limitations. Due to time and resource constraints, we were unable to perform Western blot experiments. We recognize that Western blot can provide protein-level validation, which would have further strengthened our findings, particularly regarding VNN1 expression. Additionally, our study was primarily based on bioinformatics analysis and publicly available datasets. While this approach allows for comprehensive data mining, it may not fully capture the complex biological interactions *in vivo*. Future studies involving *in vitro* and *in vivo* experiments would be valuable to validate our findings and explore the mechanistic details of VNN1’s role in COVID-19 progression. Furthermore, our molecular docking analyses, while promising, require experimental validation to confirm the efficacy of the identified compounds in modulating VNN1 activity. Lastly, the retrospective nature of our study and the potential for confounding factors in the datasets used may limit the generalizability of our results. Prospective clinical studies would be necessary to fully establish the clinical utility of VNN1 as a biomarker or therapeutic target in COVID-19.

In conclusion, our research elucidates the complex interplay between TB and COVID-19, highlighting shared mechanisms and identifying VNN1 as a key factor in disease progression. The identification of potential drugs targeting VNN1 sets the stage for future therapeutic interventions aimed at mitigating the severity of COVID-19. Despite the limitations, our findings provide valuable insights and a strong foundation for future research in this critical area of public health.

## Data Availability

The datasets presented in this study can be found in online repositories. The names of the repository/repositories and accession number(s) can be found in the article/**Supplementary Material**.
