# Supplementary material for: Exploring the shared pathogenic mechanisms of tuberculosis and COVID-19: emphasizing the role of VNN1 in severe COVID-19
Source: Front Cell Infect Microbiol. 2024 Nov 21;14:1453466. doi: 10.3389/fcimb.2024.1453466 (PMC11618882; doi:10.3389/fcimb.2024.1453466)
Supplement: Supplementary file 1 [file DataSheet1.pdf]

GSE157103	GPL24676
GSE171110	GPL16791
GSE217948	GPL24676
GSE164805	GPL26963
GSE152418	GPL24676
GSE19491	GPL6947
GSE157344	GPL18573

45Mild COVID19 55SCOVID19 26Contral
44COVID19 10Contral
333COVID19 63Contral
5Mild COVID19 5SCOVID19 5Contral
17COVID19 17Contral
54 active tuberculosis patients and 36 healthy Contral
20 samples from critically ill COVID-19 patients and 6 peripheral
136 blood samples from healthy Contral
