# Supplementary material for: Exploring the shared pathogenic mechanisms of tuberculosis and COVID-19: emphasizing the role of VNN1 in severe COVID-19
Source: Front Cell Infect Microbiol. 2024 Nov 21;14:1453466. doi: 10.3389/fcimb.2024.1453466 (PMC11618882; doi:10.3389/fcimb.2024.1453466)
Supplement: Supplementary file 2 [file DataSheet2.pdf]

Gene Symbol	Gene	Forward Primer (5’-3’)
VNN1	Vanin 1	tcctgaggtgttgctgagtg

Reverse Primer (5'-3')	UPL Probe	Accession Number
agcgtccgtcagttgacac	80	NM_004666
