# Supplementary material for: Exploring the shared pathogenic mechanisms of tuberculosis and COVID-19: emphasizing the role of VNN1 in severe COVID-19
Source: Front Cell Infect Microbiol. 2024 Nov 21;14:1453466. doi: 10.3389/fcimb.2024.1453466 (PMC11618882; doi:10.3389/fcimb.2024.1453466)
Supplement: Supplementary file 3 [file DataSheet3.pdf]

<table>
<tr><th>TB</th><th>COVID-19</th><th>TB|COVID19</th></tr>
<tr><td>PSME2</td><td>RNA28SN4</td><td>GBP5</td></tr>
<tr><td>GBP1P1</td><td>RNA5S1</td><td>STAT1</td></tr>
<tr><td>TAP1</td><td>RNA5S10</td><td>GBP1</td></tr>
<tr><td>GBP2</td><td>RNA5S11</td><td>GBP4</td></tr>
<tr><td>UBE2L6</td><td>RNA5S12</td><td>EPSTI1</td></tr>
<tr><td>VAMP5</td><td>RNA5S13</td><td>SAMD9L</td></tr>
<tr><td>PSMB9</td><td>RNA5S14</td><td>XAF1</td></tr>
<tr><td>WDFY1</td><td>RNA5S15</td><td>STAT2</td></tr>
<tr><td>FBXO6</td><td>RNA5S16</td><td>TRIM22</td></tr>
<tr><td>TAP2</td><td>RNA5S17</td><td>PARP9</td></tr>
<tr><td>IFI35</td><td>RNA5S2</td><td>XRN1</td></tr>
<tr><td>HLA-F</td><td>RNA5S3</td><td>IFI44</td></tr>
<tr><td>PSMB8</td><td>RNA5S4</td><td>BATF2</td></tr>
<tr><td>PSTPIP2</td><td>RNA5S5</td><td>IFI44L</td></tr>
<tr><td>LAP3</td><td>RNA5S6</td><td>RTP4</td></tr>
<tr><td>VPS9D1</td><td>RNA5S7</td><td>OAS3</td></tr>
<tr><td>SRBD1</td><td>RNA5S8</td><td>RNF213</td></tr>
<tr><td>SP140</td><td>HBA2</td><td>IFI6</td></tr>
<tr><td>LOC100419[illegible]</td><td>NAPRT</td><td>PARP14</td></tr>
<tr><td>ACTA2</td><td>ZNF524</td><td>IFIH1</td></tr>
<tr><td>CNIH4</td><td>S1PR4</td><td>HERC5</td></tr>
<tr><td>GCH1</td><td>TSPO</td><td>DDX60</td></tr>
<tr><td>DESI1</td><td>HBA1</td><td>OAS1</td></tr>
<tr><td>ASPHD2</td><td>ELOB</td><td>ZBP1</td></tr>
<tr><td>SEPT4</td><td>MCRIP1</td><td>EIF2AK2</td></tr>
<tr><td>GYG1</td><td>NDUFB7</td><td>OAS2</td></tr>
<tr><td>AIM2</td><td>ATP5F1D</td><td>BTN3A1</td></tr>
<tr><td>IRF1</td><td>CRIP2</td><td>OASL</td></tr>
<tr><td>PSMB10</td><td>NDUFS7</td><td>GALM</td></tr>
<tr><td>DTNBP1</td><td>DPP7</td><td>RSAD2</td></tr>
<tr><td>MT1A</td><td>TPPP3</td><td>NCOA7</td></tr>
<tr><td>PSME1</td><td>RNA5S9</td><td>SAMD9</td></tr>
<tr><td>MT2A</td><td>CD7</td><td>LY6E</td></tr>
<tr><td>MICB</td><td>CERKL</td><td>FAM8A1</td></tr>
<tr><td>WARS</td><td>HBB</td><td>TRIM5</td></tr>
<tr><td>STOM</td><td>TYMS</td><td>DDX58</td></tr>
<tr><td>NUCB1</td><td>RRM2</td><td>B4GALT5</td></tr>
<tr><td>EXOG</td><td>CDK1</td><td>IFIT5</td></tr>
<tr><td>RHBDF2</td><td>PCLAF</td><td>IFIT3</td></tr>
<tr><td>APOL2</td><td>CCNA2</td><td>SAP30</td></tr>
<tr><td>MOV10</td><td>NUSAP1</td><td>FUT7</td></tr>
<tr><td>DYNLT1</td><td>UBE2C</td><td>HNRNPLL</td></tr>
<tr><td>TRAFD1</td><td>ZWINT</td><td>MX1</td></tr>
<tr><td>TIMM10</td><td>CCNB1</td><td>FRMD3</td></tr>
<tr><td>NUB1</td><td>CCNB2</td><td>VNN1</td></tr>
<tr><td>RARRES3</td><td>CDC45</td><td>CREBRF</td></tr>
<tr><td>SQRDL</td><td>NCAPG</td><td>IRAK2</td></tr>
<tr><td>CASP1</td><td>MCM6</td><td>IFIT2</td></tr>
<tr><td>PSMA4</td><td>TOP2A</td><td>SCD</td></tr>
<tr><td>DUSP3</td><td>CDC6</td><td>DTX3L</td></tr>
</table>

MTHFD2	TPX2	IFIT1
KIF1B	UBE2T	ABCA1
MRPL44	MCM4	RNF169
LYRM1	MND1	APP
ZC3HAV1	CDCA5	GMNN
TAPBP	NDC80	
CASP4	DEPDC1B	
GSTO1	BUB1	
IFI16	DLGAP5	
EIF4E3	KIF11	
SMARCD3	JCHAIN	
SLK	HJURP	
BST2	MKI67	
C1GALT1	STMN1	
OBFC1	HMMR	
POLB	IGHG1	
GPR65	MYBL2	
IFITM3	CENPU	
ZNF438	PTTG1	
LGALS8	GINS2	
FAM111A	CDC20	
POMP	TXNDC5	
C4orf32	SPAG5	
AFF1	BIRC5	
VCPIP1	PRC1	
RHOT1	CEP55	
SCO2	GGH	
HIST1H2BK	KIF2C	
NMI	KIFC1	
METTL22	TNFRSF17	
BTN3A2	SHCBP1	
SP110	CDKN3	
ATG3	NUF2	
RBMS1	TCF19	
FYB	DHCR24	
LHFPL2	TENT5C	
GADD45B	MCM2	
WSB2	TROAP	
GLRX	CD38	
ZDHHC12	IGHG3	
CARD16	PALM2AKAP2	
TRIM21	IFI27	
SLC2A3	AURKB	
ACOT9	FEN1	
PANK2	ATAD2	
NT5C3A	E2F1	
ISG15	MZB1	
DECR1	H4C8	
CYB561	SPATS2	
IFITM1	H2BC11	
HLA-H	H2BC8	

CALCOCO2	C12orf75
PSMB3	TK1
MTPN	IGLL5
TDRD7	DHFR
IRF9	TRAM2
SBNO2	SLC2A5
PML	ELL2
C5	RPH3A
FAM160B1	LMAN1
MR1	UCHL1
TBK1	PTX3
ATF6	EGF
SNTB1	CHAC2
FKBP5	ZCCHC2
TMEM167A	CENPK
MSL3	GLDC
FAM26F	IGLC2
IGF2BP3	UAP1
ETV6	IFI27L1
HLA-A	POU2AF1
SPPL2A	IGLC1
TXN	H2BC17
BATF	ABLIM3
KREMEN1	CEP97
IRF7	IGLC3
LMNB1	ITM2C
MVP	PRRG4
TNFSF10	IRF4
BAZ1A	PATL1
CHMP5	SLA2
RBCK1	H2BC7
PHTF1	GPRC5D
IL16	CEACAM6
HDAC4	FKBP11
OSM	C18orf25
PFKFB3	PLAAT2
TPP1	SPARC
PDK3	TARM1
MICU1	H2BC9
WSB1	IGKC
LHX6	WLS
SNX3	PCGF5
AKIRIN2	MME
PTPN1	VNN3
UBQLN2	SLC38A2
MFSD14B	HSP90B1
LPCAT3	MMD
PARP11	TNFRSF13B
PARP12	HSPA13
GBA	CEACAM8
STAT3	CYP4F3

PATE2	OSGIN2
H2AFJ	CMPK2
MAX	TCN1
JAK2	APOL6
PLP2	LPIN2
ACSL4	IGHG2
IGFLR1	SEL1L3
NLRC5	LAX1
HCG4	SEC11C
NPC2	H2BC4
NFKBIB	ITGB3
ANKDD1A	NBPF10
PGS1	H2BC5
CDS2	MYZAP
BLOC1S1	NBPF14
CNDP2	SMTNL1
EIF1B	BTN3A3
ADAR	CD24
LTA4H	ELF1
UPP1	NBPF19
HELZ2	EDEM3
RMI2	RNASE2
CEACAM1	IGHA1
BCL3	PRKAR2B
GPR160	TMEM123
ERP27	ADAM17
ZNF684	DERL3
CYB5R3	CCNA1
SEC24D	F13A1
EPG5	DEFA4
VAMP3	MPO
POC1B	MMP8
PARP8	IGFL4
DPYD	SMCHD1
LACTB	BPI
SRPK1	PELI1
TRIM38	LTF
ANKRD13A	IGHG4
REC8	APOBEC3B
MLKL	DEFA1
NBN	LRRK2
NSMAF	RGS18
NPTN	OLR1
C1GALT1C1	NEK7
TFG	USP18
SHISA5	APOBEC3A_B
LTB4R	MED13L
ATOX1	MEGF9
SF3B6	RNASE3
TRANK1	CSGALNACT1
CD55	RNASE1

SAMSN1	CSGALNACT2
DPH3	MS4A3
STK3	SNURF
ERICH1	HERC3
PRCP	GBP3
CDIPT	CPEB4
YIPF1	SLFN5
SH3GLB1	CD274
N4BP2L1	FCGR3B
LYSMD2	RESF1
C4orf3	TUBB1
EXOC6	SEPTIN4
C9orf72	OTOF
EIF4G3	CARD17
ELOVL5	HERC6
CD58	CTSG
APH1B	SERPINB10
MSRB2	OLFM4
RAB1B	CRISP3
C20orf24	DEFA1B
HEBP1	SERPING1
PIK3AP1	MACIR
HOXC11	TPST1
SOS1	CAMP
HPSE	DEFA3
MTF1	IGLC7
TMEM165	LCN2
TYMP	ETV7
CHSY1	SIGLEC1
GNS	IGHM
STX11	CHIT1
PLAC8	IL18R1
RTN4	
ADRA1D	
RRBP1	
PSMA3	
EVI2A	
GLRX2	
AFTPH	
TTC32	
PHF21A	
CDK5RAP2	
MYD88	
SMIM14	
U2AF1	
HLA-G	
TOR1B	
TCTN1	
NXT2	
PDE4B	
USB1	

CMC2
IDI1
SIAH2
CTSA
LYPLA1
STOML1
DUSP5
PSMB2
FBXL5
FURIN
RNF149
HIST2H2AC
N4BP2L2
RABGAP1L
TESK2
FANCL
KIAA0355
SELL
CMAHP
RIPK2
SLC9A8
HIF1A
PLIN3
LY96
OS9
IL4R
CAPZA1
RELB
SLC4A1AP
TFE3
CLTC
SNX10
AGTRAP
PRPS2
GLMP
RSPO3
CIR1
ZNF439
ADCY3
GNG5
DHRS12
FNDC3A
MFSD13A
ZCCHC6
TNFSF13B
RIOK3
CD151
CD79B
APOL3
CKLF
HIST1H2BD

MXD3
HIST1H4K
HIST2H2AA3
CLEC2B
RBBP8
SLC26A8
GABARAPL2
AKAP13
RUBCN
SORT1
GM2A
SIPA1L1
GNA13
MAPK14
UBOX5
GNG10
TIMM8B
HNRNPH2
FAM49B
CD74
HAUS4
RAB24
MAP3K8
RNF13
TSTA3
DENND5A
NOTCH2NL
ISG20
CREG1
NOD1
NFKBIZ
ARSB
SIPA1L2
PSENEN
C1RL
FCER1G
SRGN
H3F3AP4
PLA2G4C
EAF1
LRP10
IFITM2
TRIM25
PLEK
LINC00999
NFIL3
LEPR
TRAPPC2B
DBI
FAM209B
GCLC

NABP1
HMGB2
EPB41L3
SPCS3
HLA-DMB
TMEM185B
CTBS
GIMAP2
SAT1
PASK
ITPRIPL2
NOTCH2
NKIRAS1
CYB5R4
TLR1
HIST1H1C
DENND1A
DIRC2
UHMK1
DNAJC3
CDK5R1
RNASEL
SCARB2
GNG7
ILK
SLC2A14
KLHL2
HINT3
IFNAR2
GCNT1
C1orf116
RNF19B
C9orf66
IL37
BRI3
TMEM50B
PRMT2
SAMHD1
ASAP1
GRB2
ARL5B
LOC107987345
BLVRA
C19orf12
PFKFB2
RNF135
PGRMC1
ZSCAN18
RALB
SRC
CD300A

TMOD3
VIPR1
NFAT5
SOD2
TSHZ3
RHOC
TPM4
CISD2
FTH1P3
SLC4A7
SULT1B1
PHF11
YOD1
DRAM1
ATF5
SLC30A1
LGALS9
SP100
CETP
GRAP
DGKA
NUMB
PGP
BCL6
IFI27L2
NFXL1
CNFN
HIST1H3D
RAB40B
TICAM2
FBXO38
DSE
IL17RA
VAMP4
HLA-DRB6
S100A11
FTH1
LILRA6
BRSK1
HLA-DRA
LMO2
VASP
ICA1
KRT72
RAB33B
THEMIS2
FOSB
SIRPB1
SCAND1
CD79A
ZAK

RRAGD
CTSB
DIP2A
ATF6B
PRDM1
YIPF2
HLA-DRB4
MARK2
GRN
KLHDC8B
IFNAR1
LAMP2
MSRB1
FAM129A
SGK223
CEBPB
CENPN
HIST2H2BE
TUBA3D
ANP32A
GPR183
CHURC1
CMTM1
TMEM140
ITGAM
CEP19
ERAP2
EMILIN2
RBPMS2
BPGM
