# Supplementary material for: Exploring the shared pathogenic mechanisms of tuberculosis and COVID-19: emphasizing the role of VNN1 in severe COVID-19
Source: Front Cell Infect Microbiol. 2024 Nov 21;14:1453466. doi: 10.3389/fcimb.2024.1453466 (PMC11618882; doi:10.3389/fcimb.2024.1453466)
Supplement: Supplementary file 4 [file DataSheet4.pdf]

ONTOLOGY	ID	Description	GeneRatio	BgRatio
BP	GO:0051607	defense response to virus	26/52	290/18800
BP	GO:0140546	defense response to symbiont	26/52	291/18800
BP	GO:0009615	response to virus	27/52	392/18800
BP	GO:0048525	negative regulation of viral process	13/52	91/18800
BP	GO:0050792	regulation of viral process	14/52	159/18800
BP	GO:1903900	regulation of viral life cycle	13/52	139/18800
BP	GO:0045071	negative regulation of viral genome replication	10/52	57/18800
BP	GO:0045069	regulation of viral genome replication	10/52	87/18800
BP	GO:0016032	viral process	15/52	418/18800
BP	GO:0002831	regulation of response to biotic stimulus	14/52	351/18800
BP	GO:0019058	viral life cycle	13/52	317/18800
BP	GO:0019079	viral genome replication	10/52	134/18800
BP	GO:0034340	response to type I interferon	8/52	64/18800
BP	GO:0071357	cellular response to type I interferon	7/52	58/18800
BP	GO:0060759	regulation of response to cytokine stimulus	9/52	178/18800
BP	GO:0098586	cellular response to virus	7/52	85/18800
BP	GO:0060337	type I interferon signaling pathway	6/52	56/18800
BP	GO:0032481	positive regulation of type I interferon production	6/52	58/18800
BP	GO:0002221	pattern recognition receptor signaling pathway	8/52	176/18800
BP	GO:0039528	cytoplasmic pattern recognition receptor signaling pat	5/52	34/18800
BP	GO:0050688	regulation of defense response to virus	6/52	74/18800
BP	GO:0032728	positive regulation of interferon-beta production	5/52	39/18800
BP	GO:0019221	cytokine-mediated signaling pathway	11/52	486/18800
BP	GO:0140374	antiviral innate immune response	4/52	17/18800
BP	GO:0032479	regulation of type I interferon production	6/52	98/18800
BP	GO:0032606	type I interferon production	6/52	98/18800
BP	GO:0032069	regulation of nuclease activity	4/52	21/18800
BP	GO:0001959	regulation of cytokine-mediated signaling pathway	7/52	166/18800
BP	GO:0032760	positive regulation of tumor necrosis factor productior	6/52	103/18800
BP	GO:1903557	positive regulation of tumor necrosis factor superfami	6/52	107/18800
BP	GO:0032608	interferon-beta production	5/52	57/18800
BP	GO:0032648	regulation of interferon-beta production	5/52	57/18800
BP	GO:0001819	positive regulation of cytokine production	10/52	475/18800
BP	GO:0002753	cytoplasmic pattern recognition receptor signaling pat	5/52	60/18800
BP	GO:0002764	immune response-regulating signaling pathway	10/52	482/18800
BP	GO:0039529	RIG-I signaling pathway	4/52	27/18800
BP	GO:0060760	positive regulation of response to cytokine stimulus	5/52	64/18800
BP	GO:0002230	positive regulation of defense response to virus by hos	4/52	31/18800
BP	GO:0045088	regulation of innate immune response	7/52	231/18800
BP	GO:0039530	MDA-5 signaling pathway	3/52	11/18800
BP	GO:0050691	regulation of defense response to virus by host	4/52	42/18800
BP	GO:0060338	regulation of type I interferon-mediated signaling path	4/52	42/18800
BP	GO:0035563	positive regulation of chromatin binding	3/52	14/18800
BP	GO:0032640	tumor necrosis factor production	6/52	182/18800
BP	GO:0032680	regulation of tumor necrosis factor production	6/52	182/18800
BP	GO:0071706	tumor necrosis factor superfamily cytokine productior	6/52	187/18800
BP	GO:1903555	regulation of tumor necrosis factor superfamily cytokii	6/52	187/18800
BP	GO:0071360	cellular response to exogenous dsRNA	3/52	18/18800
BP	GO:0071346	cellular response to interferon-gamma	5/52	118/18800

BP	GO:0035455	response to interferon-alpha	3/52	20/18800
BP	GO:0060339	negative regulation of type I interferon-mediated signa	3/52	20/18800
BP	GO:0032727	positive regulation of interferon-alpha production	3/52	21/18800
BP	GO:0071359	cellular response to dsRNA	3/52	23/18800
BP	GO:0035561	regulation of chromatin binding	3/52	24/18800
BP	GO:0032722	positive regulation of chemokine production	4/52	70/18800
BP	GO:0034341	response to interferon-gamma	5/52	140/18800
BP	GO:0060333	interferon-gamma-mediated signaling pathway	3/52	25/18800
BP	GO:0045824	negative regulation of innate immune response	4/52	73/18800
BP	GO:0032607	interferon-alpha production	3/52	27/18800
BP	GO:0032647	regulation of interferon-alpha production	3/52	27/18800
BP	GO:0051092	positive regulation of NF-kappaB transcription factor a	5/52	154/18800
BP	GO:0051091	positive regulation of DNA-binding transcription factor	6/52	265/18800
BP	GO:0035456	response to interferon-beta	3/52	32/18800
BP	GO:0001960	negative regulation of cytokine-mediated signaling pat	4/52	87/18800
BP	GO:0060761	negative regulation of response to cytokine stimulus	4/52	92/18800
BP	GO:0002833	positive regulation of response to biotic stimulus	5/52	180/18800
BP	GO:0032642	regulation of chemokine production	4/52	97/18800
BP	GO:0032602	chemokine production	4/52	98/18800
BP	GO:0046596	regulation of viral entry into host cell	3/52	43/18800
BP	GO:0062208	positive regulation of pattern recognition receptor sign	3/52	44/18800
BP	GO:0002832	negative regulation of response to biotic stimulus	4/52	113/18800
BP	GO:0035457	cellular response to interferon-alpha	2/52	10/18800
BP	GO:1900246	positive regulation of RIG-I signaling pathway	2/52	10/18800
BP	GO:0043330	response to exogenous dsRNA	3/52	49/18800
BP	GO:0052372	modulation by symbiont of entry into host	3/52	50/18800
BP	GO:0140289	protein mono-ADP-ribosylation	2/52	12/18800
BP	GO:0043331	response to dsRNA	3/52	56/18800
BP	GO:0043903	regulation of biological process involved in symbiotic i	3/52	56/18800
BP	GO:0051098	regulation of binding	6/52	366/18800
BP	GO:0001961	positive regulation of cytokine-mediated signaling path	3/52	57/18800
BP	GO:0070555	response to interleukin-1	4/52	141/18800
BP	GO:0071639	positive regulation of monocyte chemotactic protein-1	2/52	15/18800
BP	GO:0030522	intracellular receptor signaling pathway	5/52	264/18800
BP	GO:0060330	regulation of response to interferon-gamma	2/52	16/18800
BP	GO:0060334	regulation of interferon-gamma-mediated signaling pa	2/52	16/18800
BP	GO:2001034	positive regulation of double-strand break repair via n	2/52	16/18800
BP	GO:0072567	chemokine (C-X-C motif) ligand 2 production	2/52	18/18800
BP	GO:2000341	regulation of chemokine (C-X-C motif) ligand 2 product	2/52	18/18800
BP	GO:0044403	biological process involved in symbiotic interaction	5/52	286/18800
BP	GO:0007249	I-kappaB kinase/NF-kappaB signaling	5/52	288/18800
BP	GO:0051099	positive regulation of binding	4/52	171/18800
BP	GO:0007259	receptor signaling pathway via JAK-STAT	4/52	173/18800
BP	GO:0032103	positive regulation of response to external stimulus	6/52	442/18800
BP	GO:0039535	regulation of RIG-I signaling pathway	2/52	20/18800
BP	GO:0051090	regulation of DNA-binding transcription factor activity	6/52	452/18800
BP	GO:0050777	negative regulation of immune response	4/52	179/18800
BP	GO:0071605	monocyte chemotactic protein-1 production	2/52	21/18800
BP	GO:0071637	regulation of monocyte chemotactic protein-1 product	2/52	21/18800
BP	GO:0097696	receptor signaling pathway via STAT	4/52	184/18800

BP	GO:0046597	negative regulation of viral entry into host cell	2/52	22/18800
BP	GO:0051701	biological process involved in interaction with host	4/52	191/18800
BP	GO:0035458	cellular response to interferon-beta	2/52	24/18800
BP	GO:0039531	regulation of viral-induced cytoplasmic pattern recogn	2/52	24/18800
BP	GO:0043393	regulation of protein binding	4/52	196/18800
BP	GO:0032682	negative regulation of chemokine production	2/52	26/18800
BP	GO:0042832	defense response to protozoan	2/52	26/18800
BP	GO:1903901	negative regulation of viral life cycle	2/52	26/18800
BP	GO:0032755	positive regulation of interleukin-6 production	3/52	97/18800
BP	GO:0001562	response to protozoan	2/52	27/18800
BP	GO:2001032	regulation of double-strand break repair via nonhomol	2/52	29/18800
BP	GO:0070498	interleukin-1-mediated signaling pathway	2/52	30/18800
BP	GO:0062207	regulation of pattern recognition receptor signaling pa	3/52	106/18800
BP	GO:0042742	defense response to bacterium	5/52	364/18800
BP	GO:2000779	regulation of double-strand break repair	3/52	110/18800
BP	GO:0071347	cellular response to interleukin-1	3/52	111/18800
BP	GO:0006471	protein ADP-ribosylation	2/52	35/18800
BP	GO:0019320	hexose catabolic process	2/52	35/18800
BP	GO:0000209	protein polyubiquitination	4/52	244/18800
BP	GO:0002224	toll-like receptor signaling pathway	3/52	123/18800
BP	GO:0010742	macrophage derived foam cell differentiation	2/52	38/18800
BP	GO:0046365	monosaccharide catabolic process	2/52	39/18800
BP	GO:0090077	foam cell differentiation	2/52	39/18800
BP	GO:0043122	regulation of I-kappaB kinase/NF-kappaB signaling	4/52	254/18800
BP	GO:0014074	response to purine-containing compound	3/52	134/18800
BP	GO:0002683	negative regulation of immune system process	5/52	425/18800
BP	GO:0031348	negative regulation of defense response	4/52	268/18800
BP	GO:0045089	positive regulation of innate immune response	3/52	140/18800
BP	GO:0042551	neuron maturation	2/52	45/18800
BP	GO:0046718	viral entry into host cell	3/52	146/18800
BP	GO:0051053	negative regulation of DNA metabolic process	3/52	148/18800
BP	GO:0031349	positive regulation of defense response	4/52	289/18800
BP	GO:0006282	regulation of DNA repair	3/52	152/18800
BP	GO:0016052	carbohydrate catabolic process	3/52	152/18800
BP	GO:0044409	entry into host	3/52	153/18800
BP	GO:0051052	regulation of DNA metabolic process	5/52	472/18800
BP	GO:1903202	negative regulation of oxidative stress-induced cell dea	2/52	54/18800
BP	GO:0070534	protein K63-linked ubiquitination	2/52	57/18800
BP	GO:0002218	activation of innate immune response	2/52	59/18800
BP	GO:0032635	interleukin-6 production	3/52	172/18800
BP	GO:0032675	regulation of interleukin-6 production	3/52	172/18800
MF	GO:0003725	double-stranded RNA binding	9/53	75/18410
MF	GO:0070566	adenylyltransferase activity	3/53	30/18410
MF	GO:0003727	single-stranded RNA binding	4/53	87/18410
MF	GO:0003714	transcription corepressor activity	5/53	191/18410
MF	GO:0097677	STAT family protein binding	2/53	12/18410
MF	GO:0003712	transcription coregulator activity	7/53	497/18410
MF	GO:0003724	RNA helicase activity	3/53	77/18410
MF	GO:0004842	ubiquitin-protein transferase activity	6/53	433/18410
MF	GO:1990404	NAD+-protein ADP-ribosyltransferase activity	2/53	20/18410

MF	GO:0008186	ATP-dependent activity, acting on RNA	3/53	79/18410
MF	GO:0019787	ubiquitin-like protein transferase activity	6/53	458/18410
MF	GO:0003950	NAD+ ADP-ribosyltransferase activity	2/53	23/18410
MF	GO:0061630	ubiquitin protein ligase activity	5/53	316/18410
MF	GO:0044389	ubiquitin-like protein ligase binding	5/53	317/18410
MF	GO:0140297	DNA-binding transcription factor binding	6/53	470/18410
MF	GO:0061659	ubiquitin-like protein ligase activity	5/53	329/18410
MF	GO:0061629	RNA polymerase II-specific DNA-binding transcription f	5/53	348/18410
MF	GO:0005525	GTP binding	5/53	379/18410
MF	GO:0019001	guanyl nucleotide binding	5/53	401/18410
MF	GO:0032561	guanyl ribonucleotide binding	5/53	401/18410
MF	GO:0016779	nucleotidyltransferase activity	3/53	132/18410
MF	GO:0016922	nuclear receptor binding	3/53	139/18410
MF	GO:0016757	glycosyltransferase activity	4/53	271/18410
MF	GO:0016763	pentosyltransferase activity	2/53	49/18410
MF	GO:0004386	helicase activity	3/53	155/18410
MF	GO:0016887	ATP hydrolysis activity	4/53	325/18410
KEGG	hsa05160	Hepatitis C	10/29	157/8164
KEGG	hsa05164	Influenza A	10/29	171/8164
KEGG	hsa05162	Measles	9/29	139/8164
KEGG	hsa05171	Coronavirus disease - COVID-19	9/29	232/8164
KEGG	hsa05169	Epstein-Barr virus infection	8/29	202/8164
KEGG	hsa04621	NOD-like receptor signaling pathway	7/29	184/8164
KEGG	hsa05168	Herpes simplex virus 1 infection	8/29	495/8164
KEGG	hsa04217	Necroptosis	4/29	159/8164
KEGG	hsa05161	Hepatitis B	4/29	162/8164
KEGG	hsa05165	Human papillomavirus infection	5/29	331/8164

pvalue	p.adjust	qvalue	geneID	Count
8.7401E-34	5.4777E-31	4.3535E-31	STAT1/GBP1/	26
9.5847E-34	5.4777E-31	4.3535E-31	STAT1/GBP1/	26
5.013E-32	1.9099E-29	1.518E-29	STAT1/GBP1/	27
1.7866E-19	5.1053E-17	4.0576E-17	STAT1/OAS3/	13
7.1764E-18	1.6405E-15	1.3038E-15	STAT1/TRIM2	14
5.5274E-17	1.053E-14	8.3687E-15	TRIM22/OAS3	13
4.0944E-16	6.6856E-14	5.3135E-14	OAS3/IFIH1/C	10
3.5684E-14	5.0983E-12	4.052E-12	OAS3/IFIH1/C	10
2.6452E-13	3.3594E-11	2.6699E-11	STAT1/TRIM2	15
4.5013E-13	5.145E-11	4.0891E-11	STAT1/STAT2	14
2.4532E-12	2.5491E-10	2.0259E-10	TRIM22/OAS3	13
2.9549E-12	2.8145E-10	2.2369E-10	OAS3/IFIH1/C	10
7.6694E-12	6.7432E-10	5.3593E-10	STAT1/STAT2	8
2.1971E-10	1.7937E-08	1.4256E-08	STAT1/STAT2	7
1.2942E-09	9.8619E-08	7.8379E-08	STAT2/PARP9	9
3.4079E-09	2.4345E-07	1.9349E-07	OAS3/IFI6/IFI	7
9.7125E-09	6.5303E-07	5.19E-07	STAT1/STAT2	6
1.2057E-08	7.6562E-07	6.0849E-07	STAT1/OAS3/	6
2.6646E-08	1.603E-06	1.274E-06	OAS3/IFIH1/D	8
3.4802E-08	1.9889E-06	1.5807E-06	OAS3/IFIH1/D	5
5.336E-08	2.9043E-06	2.3082E-06	STAT1/PARP9	6
7.1263E-08	3.5478E-06	2.8197E-06	OAS3/IFIH1/C	5
7.1391E-08	3.5478E-06	2.8197E-06	STAT1/STAT2	11
1.2058E-07	5.7427E-06	4.5641E-06	OAS1/DDX58	4
2.8829E-07	1.2674E-05	1.0073E-05	STAT1/OAS3/	6
2.8829E-07	1.2674E-05	1.0073E-05	STAT1/OAS3/	6
3.0076E-07	1.2732E-05	1.0119E-05	OAS3/OAS1/C	4
3.5309E-07	1.4414E-05	1.1456E-05	STAT2/PARP9	7
3.8751E-07	1.5273E-05	1.2139E-05	OAS3/IFIH1/C	6
4.857E-07	1.783E-05	1.417E-05	OAS3/IFIH1/C	6
4.9916E-07	1.783E-05	1.417E-05	OAS3/IFIH1/C	5
4.9916E-07	1.783E-05	1.417E-05	OAS3/IFIH1/C	5
5.8945E-07	2.0416E-05	1.6226E-05	STAT1/OAS3/	10
6.4703E-07	2.1752E-05	1.7288E-05	OAS3/IFIH1/D	5
6.7346E-07	2.1993E-05	1.748E-05	GBP1/OAS3/I	10
8.7118E-07	2.766E-05	2.1983E-05	OAS3/DDX60	4
8.9579E-07	2.7673E-05	2.1993E-05	PARP9/PARP1	5
1.5492E-06	4.6598E-05	3.7035E-05	STAT1/PARP9	4
3.2276E-06	9.2643E-05	7.363E-05	STAT2/PARP9	7
3.2421E-06	9.2643E-05	7.363E-05	OAS3/IFIH1/D	3
5.3885E-06	0.00014664	0.00011655	STAT1/PARP9	4
5.3885E-06	0.00014664	0.00011655	STAT2/OAS3/	4
7.1104E-06	0.00018901	0.00015022	PARP9/DTX3L	3
1.0663E-05	0.00027083	0.00021525	OAS3/IFIH1/C	6
1.0663E-05	0.00027083	0.00021525	OAS3/IFIH1/C	6
1.2442E-05	0.00030259	0.00024049	OAS3/IFIH1/C	6
1.2442E-05	0.00030259	0.00024049	OAS3/IFIH1/C	6
1.5816E-05	0.00037661	0.00029932	IFIH1/DDX58	3
1.8369E-05	0.00042848	0.00034055	STAT1/GBP1/	5

2.2009E-05	0.00049327	0.00039203	EIF2AK2/IFIT3	3
2.2009E-05	0.00049327	0.00039203	STAT2/OAS3/	3
2.5627E-05	0.00056331	0.0004477	STAT1/IFIH1/	3
3.3992E-05	0.00073307	0.00058262	IFIH1/DDX58/	3
3.8772E-05	0.00082067	0.00065224	PARP9/DTX3L	3
4.169E-05	0.0008535	0.00067834	OAS3/OAS1/E	4
4.1816E-05	0.0008535	0.00067834	STAT1/GBP1/	5
4.3973E-05	0.00088177	0.00070081	STAT1/PARP9	3
4.9187E-05	0.00096933	0.00077039	STAT2/OAS3/	4
5.5704E-05	0.00106116	0.00084338	STAT1/IFIH1/	3
5.5704E-05	0.00106116	0.00084338	STAT1/IFIH1/	3
6.5847E-05	0.00123382	0.0009806	TRIM22/EIF2/	5
8.7623E-05	0.00161537	0.00128385	TRIM22/EIF2/	6
9.3541E-05	0.00169711	0.00134881	STAT1/XAF1/	3
9.776E-05	0.00174594	0.00138762	STAT2/OAS3/	4
0.00012147	0.00213607	0.00169769	STAT2/OAS3/	4
0.00013739	0.00237933	0.00189102	PARP9/DDX6(	5
0.00014911	0.00254369	0.00202165	OAS3/OAS1/E	4
0.00015513	0.00260762	0.00207245	OAS3/OAS1/E	4
0.0002278	0.00377354	0.00299909	TRIM22/LY6E	3
0.00024399	0.00398403	0.00316638	DDX60/OASL/	3
0.0002682	0.00431768	0.00343155	STAT2/OAS3/	4
0.00033291	0.00519182	0.0041263	IFIT3/IFIT2	2
0.00033291	0.00519182	0.0041263	DDX60/OASL	2
0.00033613	0.00519182	0.0041263	IFIH1/DDX58/	3
0.00035689	0.00543896	0.00432272	TRIM22/LY6E	3
0.00048655	0.0072315	0.00574738	PARP9/PARP1	2
0.00049887	0.0072315	0.00574738	IFIH1/DDX58/	3
0.00049887	0.0072315	0.00574738	TRIM22/LY6E	3
0.00049982	0.0072315	0.00574738	PARP9/IFIT2/	6
0.00052557	0.00750902	0.00596793	PARP9/PARP1	3
0.00062081	0.00876029	0.00696241	GBP1/ZBP1/II	4
0.00076995	0.01073231	0.00852971	OAS3/OAS1	2
0.00079725	0.01097894	0.00872572	OAS3/IFIH1/D	5
0.00087838	0.01167432	0.00927839	PARP9/PARP1	2
0.00087838	0.01167432	0.00927839	PARP9/PARP1	2
0.00087838	0.01167432	0.00927839	PARP9/DTX3L	2
0.00111598	0.01449507	0.01152023	OAS3/OAS1	2
0.00111598	0.01449507	0.01152023	OAS3/OAS1	2
0.00113986	0.01463894	0.01163457	TRIM22/LY6E	5
0.00117568	0.01493117	0.01186683	STAT1/TRIM2	5
0.00127319	0.01599187	0.01270985	PARP9/DTX3L	4
0.00132896	0.01636275	0.01300461	STAT1/STAT2	4
0.00133135	0.01636275	0.01300461	PARP9/DDX6(	6
0.00138096	0.01679192	0.0133457	DDX60/OASL	2
0.00149214	0.01777049	0.01412344	TRIM22/EIF2/	6
0.00150648	0.01777049	0.01412344	STAT2/OAS3/	4
0.00152363	0.01777049	0.01412344	OAS3/OAS1	2
0.00152363	0.01777049	0.01412344	OAS3/OAS1	2
0.00166655	0.01912274	0.01519816	STAT1/STAT2	4

0.00167303	0.01912274	0.01519816	LY6E/TRIM5	2
0.00191003	0.0216155	0.01717933	TRIM22/LY6E	4
0.00199189	0.02210417	0.01756771	STAT1/OAS1	2
0.00199189	0.02210417	0.01756771	DDX60/OASL	2
0.00209837	0.02306186	0.01832885	IFIT2/DTX3L/I	4
0.00233725	0.02496702	0.01984302	OAS3/OAS1	2
0.00233725	0.02496702	0.01984302	GBP4/BATF2	2
0.00233725	0.02496702	0.01984302	LY6E/TRIM5	2
0.00245019	0.02593113	0.02060926	IFIH1/DDX58/	3
0.00251977	0.02642289	0.0210001	GBP4/BATF2	2
0.00290432	0.03017858	0.023985	PARP9/DTX3L	2
0.00310629	0.03198635	0.02542176	ZBP1/IRAK2	2
0.00315051	0.03215206	0.02555346	DDX60/OASL/	3
0.00326289	0.03300431	0.0262308	GBP4/OAS3/F	5
0.00349726	0.03506465	0.0278683	PARP9/DTX3L	3
0.00358745	0.03565614	0.02833839	GBP1/ZBP1/IF	3
0.0042115	0.04114309	0.03269925	PARP9/PARP1	2
0.0042115	0.04114309	0.03269925	GALM/FUT7	2
0.00460108	0.04456806	0.03542132	TRIM22/RNF2	4
0.00478192	0.04593055	0.03650418	OAS1/RSAD2/	3
0.00494968	0.04714568	0.03746993	STAT1/ABCA1	2
0.00520804	0.04879334	0.03877944	GALM/FUT7	2
0.00520804	0.04879334	0.03877944	STAT1/ABCA1	2
0.00530007	0.04925186	0.03914386	STAT1/TRIM2	4
0.00606516	0.05590708	0.04443322	STAT1/XRN1/	3
0.00626562	0.0572928	0.04553455	GBP1/STAT2/	5
0.0063936	0.05799911	0.0460959	STAT2/OAS3/	4
0.00684381	0.06148036	0.04886269	PARP9/ZBP1/	3
0.00688494	0.06148036	0.04886269	B4GALT5/APF	2
0.00767939	0.06804296	0.05407844	TRIM22/LY6E	3
0.00797073	0.07008114	0.05569833	XRN1/RNF16	3
0.00830056	0.07242394	0.05756031	PARP9/ZBP1/	4
0.00857285	0.07367497	0.05855459	PARP9/DTX3L	3
0.00857285	0.07367497	0.05855459	GALM/FUT7/	3
0.00872745	0.07444388	0.05916569	TRIM22/LY6E	3
0.0096294	0.08152889	0.06479664	PARP9/XRN1/	5
0.00979553	0.08232564	0.06542987	NCOA7/VNN1	2
0.01086754	0.09066863	0.07206062	RNF213/TRIM	2
0.01160975	0.096159	0.0764242	ZBP1/TRIM5	2
0.01197964	0.09780517	0.07773253	IFIH1/DDX58/	3
0.01197964	0.09780517	0.07773253	IFIH1/DDX58/	3
7.2253E-13	1.1705E-10	9.2788E-11	OAS3/IFIH1/D	9
8.6577E-05	0.00616503	0.00488716	OAS3/OAS1/C	3
0.00011417	0.00616503	0.00488716	IFIH1/DDX60/	4
0.00021857	0.00885216	0.00701731	TRIM22/PARF	5
0.00052689	0.01423451	0.01128402	PARP9/DTX3L	2
0.0005272	0.01423451	0.01128402	TRIM22/PARF	7
0.00141795	0.0240832	0.0190913	IFIH1/DDX60/	3
0.00147428	0.0240832	0.0190913	TRIM22/RNF2	6
0.00149458	0.0240832	0.0190913	PARP9/PARP1	2

0.00152668	0.0240832	0.0190913	IFIH1/DDX60/	3
0.00195851	0.0240832	0.0190913	TRIM22/RNF2	6
0.00197919	0.0240832	0.0190913	PARP9/PARP1	2
0.00211091	0.0240832	0.0190913	TRIM22/RNF2	5
0.0021401	0.0240832	0.0190913	STAT1/STAT2/	5
0.00222993	0.0240832	0.0190913	STAT1/PARP9	6
0.00251399	0.02545415	0.02017808	TRIM22/RNF2	5
0.00320029	0.03049685	0.02417554	STAT1/PARP9	5
0.00459693	0.04137235	0.0327968	GBP1/GBP4/I	5
0.00582141	0.04715341	0.03737957	GBP1/GBP4/I	5
0.00582141	0.04715341	0.03737957	GBP1/GBP4/I	5
0.00649904	0.05013547	0.03974352	OAS3/OAS1/(	3
0.00749128	0.0538184	0.04266306	STAT1/OASL/	3
0.00764088	0.0538184	0.04266306	PARP9/PARP1	4
0.00877069	0.05920216	0.04693089	PARP9/PARP1	2
0.01008129	0.06532678	0.05178601	IFIH1/DDX60/	3
0.01416387	0.0882518	0.06995919	RNF213/IFIH1	4
7.5972E-11	4.4064E-09	3.4387E-09	STAT1/STAT2/	10
1.7748E-10	5.1468E-09	4.0166E-09	STAT1/STAT2/	10
6.9543E-10	1.3445E-08	1.0492E-08	STAT1/STAT2/	9
6.3165E-08	9.1589E-07	7.1476E-07	STAT1/STAT2/	9
3.3591E-07	3.8966E-06	3.0409E-06	STAT1/STAT2/	8
2.7037E-06	2.6135E-05	2.0396E-05	STAT1/GBP1/	7
0.00023821	0.0019737	0.00154028	STAT1/STAT2/	8
0.0022508	0.01552777	0.01211786	STAT1/STAT2/	4
0.00240948	0.01552777	0.01211786	STAT1/STAT2/	4
0.00565619	0.03280591	0.02560171	STAT1/STAT2/	5
