# Supplementary material for: Exploring the shared pathogenic mechanisms of tuberculosis and COVID-19: emphasizing the role of VNN1 in severe COVID-19
Source: Front Cell Infect Microbiol. 2024 Nov 21;14:1453466. doi: 10.3389/fcimb.2024.1453466 (PMC11618882; doi:10.3389/fcimb.2024.1453466)
Supplement: Supplementary file 5 [file DataSheet5.pdf]

ZBP1 pp SOX9
ZBP1 pp SRY
ZBP1 pp FOXC1
ZBP1 pp USF2
ZBP1 pp SREBF1
ZBP1 pp SREBF2
IFIT1 pp FOXD1
IFIT1 pp FOXO3
IFIT1 pp REL
IFIT1 pp ARID3A
IFIT1 pp FOXC1
IFIT1 pp GATA2
IFIT1 pp FOXL1
IFIT1 pp MEF2A
IFIT1 pp POU2F2
IFIT1 pp NFIC
IFIT1 pp NFYA
IFIT1 pp SRF
IFIT1 pp TP53
IFIT1 pp USF2
IFIT1 pp NR3C1
IFIT1 pp EN1
PARP9 pp FOXD1
PARP9 pp FOXI1
PARP9 pp CREB1
PARP9 pp FOXC1
PARP9 pp FOXL1
PARP9 pp YY1
PARP9 pp PPARG
PARP9 pp E2F6
PARP9 pp FOXA1
CREBRF pp FOXD1
CREBRF pp FOXC1
CREBRF pp MAX
CREBRF pp USF1
CREBRF pp USF2
CREBRF pp ELK1
CREBRF pp IRF2
CREBRF pp EN1
VNN1 pp FOXD1
VNN1 pp FOXO3
VNN1 pp JUN
VNN1 pp CREB1
VNN1 pp FOXC1
VNN1 pp NFIC
VNN1 pp SRF
VNN1 pp NFKB1
VNN1 pp TP53
VNN1 pp MAX
VNN1 pp ELK1
VNN1 pp PRDM1
VNN1 pp NR3C1
VNN1 pp PAX2
EPSTI1 pp FOXI1

EPSTI1 pp FOXC1
EPSTI1 pp GATA2
EPSTI1 pp FOXL1
EPSTI1 pp YY1
EPSTI1 pp FOXA1
HNRNPLL pp NKX3-1
HNRNPLL pp FOXC1
HNRNPLL pp GATA2
HNRNPLL pp FOXL1
HNRNPLL pp MEF2A
HNRNPLL pp NFYA
HNRNPLL pp STAT3
HNRNPLL pp GATA3
HNRNPLL pp E2F1
HNRNPLL pp TP53
HNRNPLL pp MAX
TRIM5 pp NKX3-1
TRIM5 pp FOXC1
TRIM5 pp GATA2
TRIM5 pp YY1
TRIM5 pp RELA
TRIM5 pp IRF2
TRIM5 pp ESR1
TRIM5 pp RUNX2
TRIM5 pp NR3C1
IFI6 pp REL
IFI6 pp BRCA1
IFI6 pp FOS
IFI6 pp NFIC
IFI6 pp NFYA
IFI6 pp SRF
IFI6 pp PPARG
IFI6 pp TFAP2C
EIF2AK2 pp REL
EIF2AK2 pp FOXC1
EIF2AK2 pp FOXL1
EIF2AK2 pp MEF2A
EIF2AK2 pp POU2F2
EIF2AK2 pp HOXA5
EIF2AK2 pp TP53
EIF2AK2 pp USF2
EIF2AK2 pp TEAD1
EIF2AK2 pp ELK1
LY6E pp REL
LY6E pp PRRX2
LY6E pp NKX2-5
LY6E pp FOXC1
LY6E pp POU2F2
LY6E pp TFAP2A
LY6E pp NFKB1
LY6E pp RELA
LY6E pp USF2
LY6E pp TEAD1
LY6E pp PAX2

GBP1 pp STAT1
GBP1 pp SPIB
GBP1 pp GATA2
GBP1 pp ELK4
GBP1 pp ESR1
GBP1 pp NR3C1
GBP1 pp EN1
IFIT2 pp NR2F1
IFIT2 pp FOXC1
IFIT2 pp GATA2
IFIT2 pp TEAD1
APP pp NR2F1
APP pp ZNF354C
APP pp FOXC1
APP pp GATA2
APP pp YY1
APP pp POU2F2
APP pp NFIC
APP pp NFYA
APP pp SRF
APP pp PPARG
APP pp HINFP
APP pp TFAP2A
APP pp RELA
APP pp SREBF1
APP pp ESR1
APP pp TP63
OAS2 pp NR2F1
OAS2 pp CEBPB
OAS2 pp FOS
OAS2 pp JUN
OAS2 pp CREB1
OAS2 pp FOXC1
OAS2 pp PPARG
OAS2 pp STAT3
OAS2 pp HINFP
OAS2 pp RELA
OAS2 pp TP53
OAS2 pp SREBF1
OAS2 pp SREBF2
SAMD9 pp STAT1
SAMD9 pp ZNF354C
SAMD9 pp FOXC1
SAMD9 pp GATA2
SAMD9 pp POU2F2
SAMD9 pp NFIC
SAMD9 pp HNF4A
SAMD9 pp SREBF1
FUT7 pp ZNF354C
FUT7 pp HINFP
FUT7 pp TFAP2A
FUT7 pp NFKB1
FUT7 pp RELA
FUT7 pp TP53

FUT7 pp TFAP2C
IFI44L pp ARID3A
IFI44L pp NOBOX
IFI44L pp PRRX2
IFI44L pp NKX2-5
IFI44L pp JUN
IFI44L pp YY1
IFI44L pp NR3C1
PARP14 pp NKX3-2
PARP14 pp FOXC1
PARP14 pp GATA2
PARP14 pp SRF
PARP14 pp STAT3
PARP14 pp HINFP
PARP14 pp SREBF1
PARP14 pp NR3C1
PARP14 pp EN1
B4GALT5 pp BRCA1
B4GALT5 pp CEBPB
B4GALT5 pp FOS
B4GALT5 pp JUN
B4GALT5 pp FOXC1
B4GALT5 pp GATA2
B4GALT5 pp YY1
B4GALT5 pp SREBF2
B4GALT5 pp NRF1
B4GALT5 pp ELF5
MX1 pp BRCA1
MX1 pp JUN
MX1 pp FOXC1
MX1 pp GATA2
MX1 pp MEF2A
MX1 pp POU2F2
MX1 pp TFAP2A
MX1 pp USF2
MX1 pp SREBF1
MX1 pp TP63
MX1 pp NRF1
MX1 pp EGR1
IFIH1 pp BRCA1
IFIH1 pp CREB1
IFIH1 pp NFKB1
IFIH1 pp RELA
IFIH1 pp TFAP2C
IFIT3 pp CEBPB
IFIT3 pp JUN
IFIT3 pp FOXC1
IFIT3 pp GATA2
IFIT3 pp FOXL1
IFIT3 pp YY1
IFIT3 pp MEF2A
IFIT3 pp POU2F2
IFIT3 pp NFIC
IFIT3 pp NFYA

IFIT3 pp PPARG
IFIT3 pp ELK1
IFIT3 pp ESR1
IFIT3 pp RUNX2
FRMD3 pp CEBPB
FRMD3 pp FOXC1
FRMD3 pp GATA2
FRMD3 pp FOXL1
FRMD3 pp RELA
FRMD3 pp TP53
FRMD3 pp ELK4
OASL pp FOS
OASL pp CREB1
OASL pp FOXC1
OASL pp FOXL1
OASL pp MEF2A
OASL pp PPARG
OASL pp HNF4A
OASL pp KLF5
OASL pp RELA
OASL pp USF2
OASL pp ESR1
OASL pp FOXF2
OASL pp TP63
OASL pp FOXA1
OAS3 pp FOS
OAS3 pp JUN
OAS3 pp NFKB1
OAS3 pp RELA
OAS3 pp TEAD1
GALM pp STAT1
GALM pp JUN
GALM pp PPARG
GALM pp STAT3
GALM pp GATA3
GALM pp E2F1
GALM pp USF2
GALM pp PRDM1
GALM pp SREBF2
GALM pp PAX2
STAT2 pp JUN
STAT2 pp FOXC1
STAT2 pp GATA2
STAT2 pp YY1
STAT2 pp TFAP2A
STAT2 pp TP53
STAT2 pp ELK1
GBP4 pp JUND
GBP4 pp FOXC1
GBP4 pp GATA2
GBP4 pp YY1
GBP4 pp MEF2A
GBP4 pp POU2F2
GBP4 pp HOXA5

GBP4 pp GATA3
GBP4 pp HINFP
GBP4 pp NFKB1
GBP4 pp TP53
GBP4 pp FOXA1
RSAD2 pp STAT1
RSAD2 pp JUND
RSAD2 pp CREB1
RSAD2 pp FOXC1
RSAD2 pp GATA2
RSAD2 pp FOXL1
RSAD2 pp YY1
RSAD2 pp MEF2A
RSAD2 pp HOXA5
RSAD2 pp NFIC
RSAD2 pp HINFP
RSAD2 pp NFKB1
RSAD2 pp PRDM1
SAMD9L pp JUND
SAMD9L pp GATA2
SAMD9L pp FOXL1
SAMD9L pp NFIC
SAMD9L pp NFYA
SAMD9L pp PPARG
SAMD9L pp STAT3
SAMD9L pp HINFP
SAMD9L pp NR3C1
TRIM22 pp JUND
TRIM22 pp FOXL1
TRIM22 pp NFIC
TRIM22 pp NFYA
TRIM22 pp IRF2
TRIM22 pp RUNX2
SCD pp CREB1
SCD pp FOXC1
SCD pp GATA2
SCD pp FOXL1
SCD pp NFIC
SCD pp PPARG
SCD pp HNF4A
SCD pp TFAP2A
SCD pp NFKB1
SCD pp RELA
SCD pp TP53
SCD pp IRF2
FAM8A1 pp CREB1
FAM8A1 pp PPARG
FAM8A1 pp GATA3
FAM8A1 pp NFKB1
FAM8A1 pp FOXF2
STAT1 pp BTN3A1
STAT1 pp HERC5
STAT1 pp CREB1
STAT1 pp GATA2

STAT1 pp E2F1
STAT1 pp NFKB1
STAT1 pp RELA
STAT1 pp PRDM1
DTX3L pp FOXC1
DTX3L pp FOXL1
DTX3L pp MEF2A
DTX3L pp HOXA5
DTX3L pp HNF4A
DTX3L pp NR3C1
SAP30 pp FOXC1
SAP30 pp GATA2
SAP30 pp FOXL1
SAP30 pp HINFP
SAP30 pp NRF1
DDX58 pp FOXC1
DDX58 pp GATA2
DDX58 pp POU2F2
DDX58 pp NFIC
DDX58 pp USF2
DDX58 pp PRDM1
DDX58 pp SOX10
BTN3A1 pp FOXC1
BTN3A1 pp E2F1
BTN3A1 pp PRDM1
BTN3A1 pp ESR1
XAF1 pp FOXC1
XAF1 pp NFIC
XAF1 pp HNF4A
XAF1 pp RELA
XAF1 pp TFAP2C
XRN1 pp FOXC1
XRN1 pp YY1
XRN1 pp POU2F2
XRN1 pp HOXA5
XRN1 pp ELK1
XRN1 pp IRF2
XRN1 pp PRDM1
GMNN pp FOXC1
GMNN pp MEF2A
GMNN pp NRF1
DDX60 pp FOXC1
DDX60 pp GATA2
DDX60 pp MEF2A
DDX60 pp TEAD1
NCOA7 pp FOXC1
NCOA7 pp FOXL1
NCOA7 pp MEF2A
NCOA7 pp POU2F2
NCOA7 pp SRF
NCOA7 pp PPARG
NCOA7 pp STAT3
NCOA7 pp E2F1
NCOA7 pp HINFP

NCOA7 pp TFAP2A
NCOA7 pp NR3C1
NCOA7 pp EN1
NCOA7 pp SOX17
IRAK2 pp FOXC1
IRAK2 pp FOXL1
IRAK2 pp PPARG
IRAK2 pp KLF5
IRAK2 pp ESR1
IRAK2 pp FOXF2
RNF213 pp FOXC1
RNF213 pp E2F1
RNF213 pp HINFP
RNF213 pp SP1
RNF213 pp TFAP2A
RNF213 pp NFKB1
RNF213 pp TP53
RNF213 pp ESR1
RNF213 pp SREBF2
RNF213 pp NRF1
GBP5 pp FOXC1
GBP5 pp NFIC
GBP5 pp NFYA
GBP5 pp SRF
GBP5 pp NFKB1
GBP5 pp RELA
GBP5 pp TP53
GBP5 pp USF2
GBP5 pp PRDM1
GBP5 pp RUNX2
GBP5 pp NR3C1
GBP5 pp PAX2
GBP5 pp SOX5
RTP4 pp FOXC1
RTP4 pp GATA2
RTP4 pp NFIC
RTP4 pp NFYA
RTP4 pp SRF
RTP4 pp E2F1
RTP4 pp E2F6
RTP4 pp MAX
RTP4 pp USF1
RTP4 pp USF2
RTP4 pp TFAP2C
ABCA1 pp FOXC1
ABCA1 pp GATA2
ABCA1 pp HNF4A
ABCA1 pp MAX
ABCA1 pp USF1
ABCA1 pp USF2
ABCA1 pp SREBF1
IFI44 pp FOXC1
IFI44 pp MEF2A
IFI44 pp POU2F2

IFI44 pp TP53
IFI44 pp MAX
IFI44 pp USF1
IFI44 pp USF2
IFI44 pp NR3C1
OAS1 pp FOXC1
OAS1 pp GATA2
OAS1 pp FOXL1
OAS1 pp E2F1
OAS1 pp SREBF1
OAS1 pp ELK1
OAS1 pp PRDM1
HERC5 pp GATA2
HERC5 pp POU2F2
HERC5 pp NFIC
HERC5 pp GATA3
HERC5 pp HINFP
HERC5 pp SP1
HERC5 pp TP53
HERC5 pp MAX
HERC5 pp USF1
HERC5 pp USF2
HERC5 pp SREBF1
HERC5 pp ELK1
RNF169 pp GATA2
RNF169 pp NFIC
RNF169 pp USF2
RNF169 pp ELK1
RNF169 pp PAX2
IFIT5 pp IRF2
BATF2 pp ELK4
BATF2 pp TP63
