# Supplementary material for: Exploring the shared pathogenic mechanisms of tuberculosis and COVID-19: emphasizing the role of VNN1 in severe COVID-19
Source: Front Cell Infect Microbiol. 2024 Nov 21;14:1453466. doi: 10.3389/fcimb.2024.1453466 (PMC11618882; doi:10.3389/fcimb.2024.1453466)
Supplement: Supplementary file 6 [file DataSheet6.pdf]

APP pp hsa-let-7a-5p
APP pp hsa-let-7d-5p
APP pp hsa-mir-15a-5p
APP pp hsa-mir-16-5p
APP pp hsa-mir-17-5p
APP pp hsa-mir-20a-5p
APP pp hsa-mir-101-3p
APP pp hsa-mir-106a-5p
APP pp hsa-mir-196a-5p
APP pp hsa-mir-222-3p
APP pp hsa-mir-15b-5p
APP pp hsa-mir-130a-3p
APP pp hsa-mir-144-3p
APP pp hsa-mir-195-5p
APP pp hsa-mir-106b-5p
APP pp hsa-mir-302b-5p
APP pp hsa-mir-328-3p
APP pp hsa-mir-423-3p
APP pp hsa-mir-424-5p
APP pp hsa-mir-484
APP pp hsa-mir-497-5p
APP pp hsa-mir-520c-3p
APP pp hsa-mir-522-3p
APP pp hsa-mir-567
APP pp hsa-mir-579-3p
APP pp hsa-mir-583
APP pp hsa-mir-548a-3p
APP pp hsa-mir-297
APP pp hsa-let-7g-3p
APP pp hsa-mir-302d-5p
APP pp hsa-mir-424-3p
APP pp hsa-mir-490-5p
APP pp hsa-mir-500a-5p
APP pp hsa-mir-532-3p
APP pp hsa-mir-455-3p
APP pp hsa-mir-545-5p
APP pp hsa-mir-551b-5p
APP pp hsa-mir-298
APP pp hsa-mir-944
APP pp hsa-mir-1238-3p
APP pp hsa-mir-548e-3p
APP pp hsa-mir-548f-3p
APP pp hsa-mir-1276
APP pp hsa-mir-224-3p
APP pp hsa-let-7a-2-3p
APP pp hsa-mir-3120-3p
APP pp hsa-mir-3132
APP pp hsa-mir-1260b
APP pp hsa-mir-4311
APP pp hsa-mir-3606-5p
APP pp hsa-mir-3620-3p
APP pp hsa-mir-3646
APP pp hsa-mir-3662
APP pp hsa-mir-3672

APP pp hsa-mir-3688-3p
APP pp hsa-mir-3908
APP pp hsa-mir-3924
APP pp hsa-mir-548ad-3p
APP pp hsa-mir-4461
APP pp hsa-mir-4484
APP pp hsa-mir-4495
APP pp hsa-mir-4698
APP pp hsa-mir-4709-3p
APP pp hsa-mir-4786-5p
APP pp hsa-mir-5011-5p
APP pp hsa-mir-5093
APP pp hsa-mir-548ar-3p
APP pp hsa-mir-664b-3p
APP pp hsa-mir-5582-3p
APP pp hsa-mir-5584-5p
APP pp hsa-mir-5696
APP pp hsa-mir-197-5p
APP pp hsa-mir-1229-5p
APP pp hsa-mir-6073
APP pp hsa-mir-548az-3p
APP pp hsa-mir-6502-3p
APP pp hsa-mir-6504-3p
APP pp hsa-mir-190a-3p
APP pp hsa-mir-670-3p
APP pp hsa-mir-6780a-3p
APP pp hsa-mir-6838-5p
APP pp hsa-mir-6864-3p
APP pp hsa-mir-6881-3p
APP pp hsa-mir-7111-3p
STAT2 pp hsa-let-7a-5p
STAT2 pp hsa-let-7b-5p
STAT2 pp hsa-let-7c-5p
STAT2 pp hsa-let-7d-5p
STAT2 pp hsa-let-7e-5p
STAT2 pp hsa-let-7f-5p
STAT2 pp hsa-mir-25-3p
STAT2 pp hsa-mir-32-5p
STAT2 pp hsa-mir-92a-3p
STAT2 pp hsa-mir-98-5p
STAT2 pp hsa-let-7g-5p
STAT2 pp hsa-let-7i-5p
STAT2 pp hsa-mir-125a-5p
STAT2 pp hsa-mir-363-3p
STAT2 pp hsa-mir-367-3p
STAT2 pp hsa-mir-373-5p
STAT2 pp hsa-mir-335-5p
STAT2 pp hsa-mir-485-5p
STAT2 pp hsa-mir-202-3p
STAT2 pp hsa-mir-92b-3p
STAT2 pp hsa-mir-616-5p
STAT2 pp hsa-mir-548c-3p
STAT2 pp hsa-mir-658
STAT2 pp hsa-mir-25-5p

STAT2 pp hsa-mir-221-5p
STAT2 pp hsa-mir-371a-5p
STAT2 pp hsa-mir-377-5p
STAT2 pp hsa-mir-455-3p
STAT2 pp hsa-mir-665
STAT2 pp hsa-mir-1228-3p
STAT2 pp hsa-mir-1912
STAT2 pp hsa-mir-548s
STAT2 pp hsa-mir-3130-5p
STAT2 pp hsa-mir-3190-5p
STAT2 pp hsa-mir-4325
STAT2 pp hsa-mir-4266
STAT2 pp hsa-mir-3613-3p
STAT2 pp hsa-mir-1273e
STAT2 pp hsa-mir-3926
STAT2 pp hsa-mir-3929
STAT2 pp hsa-mir-4458
STAT2 pp hsa-mir-4459
STAT2 pp hsa-mir-4478
STAT2 pp hsa-mir-4482-5p
STAT2 pp hsa-mir-4419b
STAT2 pp hsa-mir-4500
STAT2 pp hsa-mir-4537
STAT2 pp hsa-mir-4668-3p
STAT2 pp hsa-mir-4695-5p
STAT2 pp hsa-mir-371b-5p
STAT2 pp hsa-mir-4779
STAT2 pp hsa-mir-1295b-5p
STAT2 pp hsa-mir-6086
STAT2 pp hsa-mir-6499-3p
STAT2 pp hsa-mir-372-5p
STAT2 pp hsa-mir-6840-3p
STAT2 pp hsa-mir-6884-5p
STAT2 pp hsa-mir-6894-5p
STAT2 pp hsa-mir-7160-5p
STAT2 pp hsa-mir-7162-3p
STAT2 pp hsa-mir-7703
STAT2 pp hsa-mir-6516-5p
STAT2 pp hsa-mir-8073
TRIM5 pp hsa-let-7a-5p
TRIM5 pp hsa-mir-218-5p
TRIM5 pp hsa-mir-190a-5p
TRIM5 pp hsa-mir-539-5p
TRIM5 pp hsa-mir-599
TRIM5 pp hsa-mir-190b
TRIM5 pp hsa-mir-548u
TRIM5 pp hsa-mir-3161
TRIM5 pp hsa-mir-3689a-5p
TRIM5 pp hsa-mir-3689b-5p
TRIM5 pp hsa-mir-3689e
TRIM5 pp hsa-mir-3689f
TRIM5 pp hsa-mir-4712-3p
TRIM5 pp hsa-mir-7161-5p
TRIM5 pp hsa-mir-8070

TRIM5 pp hsa-mir-8087
DTX3L pp hsa-let-7a-5p
DTX3L pp hsa-let-7b-5p
DTX3L pp hsa-let-7c-5p
DTX3L pp hsa-let-7d-5p
DTX3L pp hsa-let-7e-5p
DTX3L pp hsa-let-7f-5p
DTX3L pp hsa-mir-21-5p
DTX3L pp hsa-mir-98-5p
DTX3L pp hsa-mir-129-5p
DTX3L pp hsa-let-7g-5p
DTX3L pp hsa-let-7i-5p
DTX3L pp hsa-mir-485-5p
DTX3L pp hsa-mir-202-3p
DTX3L pp hsa-mir-520g-3p
DTX3L pp hsa-mir-520h
DTX3L pp hsa-mir-607
DTX3L pp hsa-mir-766-3p
DTX3L pp hsa-mir-377-5p
DTX3L pp hsa-mir-488-3p
DTX3L pp hsa-mir-455-3p
DTX3L pp hsa-mir-665
DTX3L pp hsa-mir-4252
DTX3L pp hsa-mir-4282
DTX3L pp hsa-mir-3622b-5p
DTX3L pp hsa-mir-4458
DTX3L pp hsa-mir-3689d
DTX3L pp hsa-mir-4500
DTX3L pp hsa-mir-3973
DTX3L pp hsa-mir-4738-5p
DTX3L pp hsa-mir-4793-3p
DTX3L pp hsa-mir-4795-5p
DTX3L pp hsa-mir-5009-3p
DTX3L pp hsa-mir-5197-5p
DTX3L pp hsa-mir-1247-3p
DTX3L pp hsa-mir-1277-5p
DTX3L pp hsa-mir-1273g-3p
DTX3L pp hsa-mir-3606-3p
DTX3L pp hsa-mir-6086
DTX3L pp hsa-mir-6499-3p
DTX3L pp hsa-mir-6507-3p
DTX3L pp hsa-mir-510-3p
DTX3L pp hsa-mir-6746-5p
DTX3L pp hsa-mir-6771-5p
DTX3L pp hsa-mir-6807-5p
DTX3L pp hsa-mir-6851-5p
DTX3L pp hsa-mir-6884-5p
DTX3L pp hsa-mir-6888-5p
DTX3L pp hsa-mir-7162-3p
DTX3L pp hsa-mir-7703
DTX3L pp hsa-mir-6516-5p
DTX3L pp hsa-mir-7847-3p
SCD pp hsa-let-7b-5p
SCD pp hsa-let-7e-5p

SCD pp hsa-mir-17-5p
SCD pp hsa-mir-19a-3p
SCD pp hsa-mir-19b-3p
SCD pp hsa-mir-20a-5p
SCD pp hsa-mir-22-3p
SCD pp hsa-mir-30a-5p
SCD pp hsa-mir-92a-3p
SCD pp hsa-mir-93-5p
SCD pp hsa-mir-105-5p
SCD pp hsa-mir-106a-5p
SCD pp hsa-mir-192-5p
SCD pp hsa-mir-197-3p
SCD pp hsa-mir-198
SCD pp hsa-mir-10a-5p
SCD pp hsa-mir-181a-5p
SCD pp hsa-mir-181b-5p
SCD pp hsa-mir-181c-5p
SCD pp hsa-mir-215-5p
SCD pp hsa-mir-142-5p
SCD pp hsa-mir-142-3p
SCD pp hsa-mir-149-5p
SCD pp hsa-mir-185-5p
SCD pp hsa-mir-155-5p
SCD pp hsa-mir-106b-5p
SCD pp hsa-mir-130b-3p
SCD pp hsa-mir-328-3p
SCD pp hsa-mir-331-3p
SCD pp hsa-mir-324-5p
SCD pp hsa-mir-335-5p
SCD pp hsa-mir-20b-5p
SCD pp hsa-mir-323b-5p
SCD pp hsa-mir-484
SCD pp hsa-mir-493-5p
SCD pp hsa-mir-181d-5p
SCD pp hsa-mir-526b-3p
SCD pp hsa-mir-519d-3p
SCD pp hsa-mir-505-3p
SCD pp hsa-mir-544a
SCD pp hsa-mir-558
SCD pp hsa-mir-583
SCD pp hsa-mir-605-5p
SCD pp hsa-mir-606
SCD pp hsa-mir-607
SCD pp hsa-mir-610
SCD pp hsa-mir-548c-3p
SCD pp hsa-mir-631
SCD pp hsa-mir-634
SCD pp hsa-mir-645
SCD pp hsa-mir-646
SCD pp hsa-mir-421
SCD pp hsa-mir-214-5p
SCD pp hsa-mir-143-5p
SCD pp hsa-mir-149-3p
SCD pp hsa-mir-188-3p

SCD pp hsa-mir-424-3p
SCD pp hsa-mir-455-3p
SCD pp hsa-mir-551b-5p
SCD pp hsa-mir-574-5p
SCD pp hsa-mir-625-3p
SCD pp hsa-mir-873-5p
SCD pp hsa-mir-920
SCD pp hsa-mir-944
SCD pp hsa-mir-1224-3p
SCD pp hsa-mir-1229-3p
SCD pp hsa-mir-1286
SCD pp hsa-mir-1254
SCD pp hsa-mir-1260a
SCD pp hsa-mir-1269a
SCD pp hsa-mir-1288-3p
SCD pp hsa-mir-1321
SCD pp hsa-mir-2861
SCD pp hsa-mir-3120-3p
SCD pp hsa-mir-3133
SCD pp hsa-mir-3136-5p
SCD pp hsa-mir-548v
SCD pp hsa-mir-3148
SCD pp hsa-mir-1260b
SCD pp hsa-mir-3173-3p
SCD pp hsa-mir-3188
SCD pp hsa-mir-4300
SCD pp hsa-mir-4306
SCD pp hsa-mir-4262
SCD pp hsa-mir-4280
SCD pp hsa-mir-3609
SCD pp hsa-mir-3659
SCD pp hsa-mir-3661
SCD pp hsa-mir-3671
SCD pp hsa-mir-3690
SCD pp hsa-mir-3692-3p
SCD pp hsa-mir-3908
SCD pp hsa-mir-3936
SCD pp hsa-mir-3942-5p
SCD pp hsa-mir-4426
SCD pp hsa-mir-4433a-3p
SCD pp hsa-mir-4439
SCD pp hsa-mir-548ah-5p
SCD pp hsa-mir-4459
SCD pp hsa-mir-4468
SCD pp hsa-mir-4502
SCD pp hsa-mir-4511
SCD pp hsa-mir-4513
SCD pp hsa-mir-4515
SCD pp hsa-mir-4520-3p
SCD pp hsa-mir-1269b
SCD pp hsa-mir-3140-5p
SCD pp hsa-mir-3160-5p
SCD pp hsa-mir-3177-5p
SCD pp hsa-mir-3944-5p

SCD pp hsa-mir-4638-3p
SCD pp hsa-mir-4644
SCD pp hsa-mir-4647
SCD pp hsa-mir-4658
SCD pp hsa-mir-4660
SCD pp hsa-mir-4662b
SCD pp hsa-mir-4668-5p
SCD pp hsa-mir-4668-3p
SCD pp hsa-mir-4685-5p
SCD pp hsa-mir-4699-5p
SCD pp hsa-mir-4703-5p
SCD pp hsa-mir-4709-5p
SCD pp hsa-mir-203b-5p
SCD pp hsa-mir-4712-3p
SCD pp hsa-mir-4722-5p
SCD pp hsa-mir-4728-5p
SCD pp hsa-mir-4733-5p
SCD pp hsa-mir-4739
SCD pp hsa-mir-4740-5p
SCD pp hsa-mir-499b-5p
SCD pp hsa-mir-4756-5p
SCD pp hsa-mir-4766-3p
SCD pp hsa-mir-4796-3p
SCD pp hsa-mir-5004-5p
SCD pp hsa-mir-5584-5p
SCD pp hsa-mir-5590-3p
SCD pp hsa-mir-5591-5p
SCD pp hsa-mir-5680
SCD pp hsa-mir-211-3p
SCD pp hsa-mir-3190-3p
SCD pp hsa-mir-6071
SCD pp hsa-mir-6079
SCD pp hsa-mir-6501-3p
SCD pp hsa-mir-6505-3p
SCD pp hsa-mir-6718-5p
SCD pp hsa-mir-95-5p
SCD pp hsa-mir-410-5p
SCD pp hsa-mir-494-5p
SCD pp hsa-mir-1251-3p
SCD pp hsa-mir-6726-5p
SCD pp hsa-mir-6730-5p
SCD pp hsa-mir-6731-5p
SCD pp hsa-mir-6740-5p
SCD pp hsa-mir-6748-3p
SCD pp hsa-mir-6758-5p
SCD pp hsa-mir-6760-5p
SCD pp hsa-mir-6785-5p
SCD pp hsa-mir-6790-5p
SCD pp hsa-mir-6797-5p
SCD pp hsa-mir-6811-3p
SCD pp hsa-mir-6828-3p
SCD pp hsa-mir-6833-5p
SCD pp hsa-mir-6837-5p
SCD pp hsa-mir-6855-3p

SCD pp hsa-mir-6856-5p
SCD pp hsa-mir-6857-3p
SCD pp hsa-mir-6865-3p
SCD pp hsa-mir-6882-3p
SCD pp hsa-mir-6883-5p
SCD pp hsa-mir-6884-3p
SCD pp hsa-mir-6891-5p
SCD pp hsa-mir-7113-5p
SCD pp hsa-mir-7114-5p
SCD pp hsa-mir-7154-3p
SCD pp hsa-mir-4433b-3p
SCD pp hsa-mir-7845-5p
SCD pp hsa-mir-7853-5p
SCD pp hsa-mir-8078
SCD pp hsa-mir-8085
SCD pp hsa-mir-1249-5p
IFIT5 pp hsa-let-7b-5p
IFIT5 pp hsa-mir-146a-5p
IFIT5 pp hsa-mir-155-5p
IFIT5 pp hsa-mir-106b-5p
XRN1 pp hsa-let-7b-5p
XRN1 pp hsa-mir-32-5p
XRN1 pp hsa-mir-92a-3p
XRN1 pp hsa-mir-10a-5p
XRN1 pp hsa-mir-34a-5p
XRN1 pp hsa-mir-335-5p
XRN1 pp hsa-mir-410-3p
XRN1 pp hsa-mir-92b-3p
XRN1 pp hsa-mir-4747-3p
XRN1 pp hsa-mir-5011-5p
XRN1 pp hsa-mir-1295b-3p
XRN1 pp hsa-mir-1277-5p
XRN1 pp hsa-mir-6507-5p
XRN1 pp hsa-mir-190a-3p
CREBRF pp hsa-mir-15a-5p
CREBRF pp hsa-mir-16-5p
CREBRF pp hsa-mir-19a-3p
CREBRF pp hsa-mir-19b-3p
CREBRF pp hsa-mir-26a-5p
CREBRF pp hsa-mir-26b-5p
CREBRF pp hsa-mir-93-5p
CREBRF pp hsa-mir-103a-3p
CREBRF pp hsa-mir-107
CREBRF pp hsa-mir-147a
CREBRF pp hsa-mir-15b-5p
CREBRF pp hsa-mir-124-3p
CREBRF pp hsa-mir-142-5p
CREBRF pp hsa-mir-9-3p
CREBRF pp hsa-mir-186-5p
CREBRF pp hsa-mir-195-5p
CREBRF pp hsa-mir-320a
CREBRF pp hsa-mir-155-5p
CREBRF pp hsa-mir-302a-3p
CREBRF pp hsa-mir-302b-3p

CREBRF pp hsa-mir-302c-3p
CREBRF pp hsa-mir-302d-3p
CREBRF pp hsa-mir-369-3p
CREBRF pp hsa-mir-372-3p
CREBRF pp hsa-mir-373-3p
CREBRF pp hsa-mir-381-3p
CREBRF pp hsa-mir-383-5p
CREBRF pp hsa-mir-335-5p
CREBRF pp hsa-mir-424-5p
CREBRF pp hsa-mir-323b-5p
CREBRF pp hsa-mir-484
CREBRF pp hsa-mir-497-5p
CREBRF pp hsa-mir-520e
CREBRF pp hsa-mir-519c-3p
CREBRF pp hsa-mir-520a-3p
CREBRF pp hsa-mir-526b-5p
CREBRF pp hsa-mir-519b-3p
CREBRF pp hsa-mir-520b
CREBRF pp hsa-mir-520c-3p
CREBRF pp hsa-mir-520d-3p
CREBRF pp hsa-mir-522-3p
CREBRF pp hsa-mir-519a-3p
CREBRF pp hsa-mir-503-5p
CREBRF pp hsa-mir-506-3p
CREBRF pp hsa-mir-577
CREBRF pp hsa-mir-588
CREBRF pp hsa-mir-639
CREBRF pp hsa-mir-641
CREBRF pp hsa-mir-646
CREBRF pp hsa-mir-655-3p
CREBRF pp hsa-mir-421
CREBRF pp hsa-let-7a-3p
CREBRF pp hsa-let-7b-3p
CREBRF pp hsa-let-7f-1-3p
CREBRF pp hsa-mir-100-3p
CREBRF pp hsa-mir-223-5p
CREBRF pp hsa-mir-136-3p
CREBRF pp hsa-mir-155-3p
CREBRF pp hsa-mir-340-5p
CREBRF pp hsa-mir-338-5p
CREBRF pp hsa-mir-20b-3p
CREBRF pp hsa-mir-574-5p
CREBRF pp hsa-mir-298
CREBRF pp hsa-mir-300
CREBRF pp hsa-mir-873-5p
CREBRF pp hsa-mir-374b-3p
CREBRF pp hsa-mir-1237-3p
CREBRF pp hsa-mir-320b
CREBRF pp hsa-mir-320c
CREBRF pp hsa-mir-1179
CREBRF pp hsa-mir-1184
CREBRF pp hsa-mir-1207-5p
CREBRF pp hsa-mir-1297
CREBRF pp hsa-mir-1246

CREBRF pp hsa-mir-1248
CREBRF pp hsa-mir-302e
CREBRF pp hsa-mir-320d
CREBRF pp hsa-mir-224-3p
CREBRF pp hsa-mir-449b-3p
CREBRF pp hsa-mir-711
CREBRF pp hsa-mir-3137
CREBRF pp hsa-mir-3148
CREBRF pp hsa-mir-3074-3p
CREBRF pp hsa-mir-3182
CREBRF pp hsa-mir-4310
CREBRF pp hsa-mir-4256
CREBRF pp hsa-mir-4268
CREBRF pp hsa-mir-4272
CREBRF pp hsa-mir-3617-5p
CREBRF pp hsa-mir-3679-3p
CREBRF pp hsa-mir-3685
CREBRF pp hsa-mir-3686
CREBRF pp hsa-mir-3714
CREBRF pp hsa-mir-3910
CREBRF pp hsa-mir-3935
CREBRF pp hsa-mir-374c-5p
CREBRF pp hsa-mir-4429
CREBRF pp hsa-mir-4448
CREBRF pp hsa-mir-4465
CREBRF pp hsa-mir-4524a-5p
CREBRF pp hsa-mir-4535
CREBRF pp hsa-mir-3124-3p
CREBRF pp hsa-mir-4666a-3p
CREBRF pp hsa-mir-4690-3p
CREBRF pp hsa-mir-4694-3p
CREBRF pp hsa-mir-4701-5p
CREBRF pp hsa-mir-4736
CREBRF pp hsa-mir-4752
CREBRF pp hsa-mir-4755-3p
CREBRF pp hsa-mir-4763-3p
CREBRF pp hsa-mir-4773
CREBRF pp hsa-mir-4776-5p
CREBRF pp hsa-mir-4778-3p
CREBRF pp hsa-mir-4799-5p
CREBRF pp hsa-mir-5006-5p
CREBRF pp hsa-mir-5011-5p
CREBRF pp hsa-mir-5186
CREBRF pp hsa-mir-5197-5p
CREBRF pp hsa-mir-4524b-5p
CREBRF pp hsa-mir-5580-3p
CREBRF pp hsa-mir-5582-5p
CREBRF pp hsa-mir-5590-3p
CREBRF pp hsa-mir-5685
CREBRF pp hsa-mir-5687
CREBRF pp hsa-mir-5694
CREBRF pp hsa-mir-5697
CREBRF pp hsa-mir-1277-5p
CREBRF pp hsa-mir-3190-3p

CREBRF pp hsa-mir-98-3p
CREBRF pp hsa-mir-376a-2-5p
CREBRF pp hsa-mir-758-5p
CREBRF pp hsa-mir-4750-3p
CREBRF pp hsa-mir-6074
CREBRF pp hsa-mir-6083
CREBRF pp hsa-mir-6124
CREBRF pp hsa-let-7c-3p
CREBRF pp hsa-mir-190a-3p
CREBRF pp hsa-mir-329-5p
CREBRF pp hsa-mir-410-5p
CREBRF pp hsa-mir-494-5p
CREBRF pp hsa-mir-6740-3p
CREBRF pp hsa-mir-6772-3p
CREBRF pp hsa-mir-6833-5p
CREBRF pp hsa-mir-6838-5p
CREBRF pp hsa-mir-6867-5p
CREBRF pp hsa-mir-6868-3p
CREBRF pp hsa-mir-6882-5p
CREBRF pp hsa-mir-7150
CREBRF pp hsa-mir-7157-5p
CREBRF pp hsa-mir-8066
ABCA1 pp hsa-mir-17-5p
ABCA1 pp hsa-mir-19a-3p
ABCA1 pp hsa-mir-20a-5p
ABCA1 pp hsa-mir-26a-5p
ABCA1 pp hsa-mir-26b-5p
ABCA1 pp hsa-mir-27a-3p
ABCA1 pp hsa-mir-33a-5p
ABCA1 pp hsa-mir-93-5p
ABCA1 pp hsa-mir-27b-3p
ABCA1 pp hsa-mir-128-3p
ABCA1 pp hsa-mir-106b-5p
ABCA1 pp hsa-mir-302a-3p
ABCA1 pp hsa-mir-20b-5p
ABCA1 pp hsa-mir-519d-3p
ABCA1 pp hsa-mir-613
ABCA1 pp hsa-mir-33b-5p
ABCA1 pp hsa-mir-33a-3p
ABCA1 pp hsa-mir-148a-5p
ABCA1 pp hsa-mir-33b-3p
BTN3A1 pp hsa-mir-17-5p
BTN3A1 pp hsa-mir-20a-5p
BTN3A1 pp hsa-mir-93-5p
BTN3A1 pp hsa-mir-106b-5p
BTN3A1 pp hsa-mir-324-5p
BTN3A1 pp hsa-mir-335-5p
BTN3A1 pp hsa-mir-20b-5p
BTN3A1 pp hsa-mir-519d-3p
FAM8A1 pp hsa-mir-17-5p
FAM8A1 pp hsa-mir-30a-5p
FAM8A1 pp hsa-mir-30c-5p
FAM8A1 pp hsa-mir-30d-5p
FAM8A1 pp hsa-mir-30b-5p

FAM8A1 pp hsa-mir-99b-5p
FAM8A1 pp hsa-mir-30e-5p
FAM8A1 pp hsa-mir-376c-3p
FAM8A1 pp hsa-mir-524-5p
FAM8A1 pp hsa-mir-520d-5p
FAM8A1 pp hsa-mir-590-3p
FAM8A1 pp hsa-mir-1305
FAM8A1 pp hsa-mir-2355-5p
FAM8A1 pp hsa-mir-3679-3p
FAM8A1 pp hsa-mir-4424
FAM8A1 pp hsa-mir-4775
FAM8A1 pp hsa-mir-6840-3p
FAM8A1 pp hsa-mir-7844-5p
SAMD9L pp hsa-mir-17-5p
SAMD9L pp hsa-mir-20a-5p
SAMD9L pp hsa-mir-93-5p
SAMD9L pp hsa-mir-146a-5p
SAMD9L pp hsa-mir-106b-5p
SAMD9L pp hsa-mir-20b-5p
SAMD9L pp hsa-mir-517-5p
SAMD9L pp hsa-mir-519d-3p
SAMD9L pp hsa-mir-574-5p
SAMD9L pp hsa-mir-939-5p
SAMD9L pp hsa-mir-2110
SAMD9L pp hsa-mir-3147
SAMD9L pp hsa-mir-3150a-3p
SAMD9L pp hsa-mir-3175
SAMD9L pp hsa-mir-1273e
SAMD9L pp hsa-mir-3672
SAMD9L pp hsa-mir-4425
SAMD9L pp hsa-mir-4455
SAMD9L pp hsa-mir-5192
SAMD9L pp hsa-mir-873-3p
SAMD9L pp hsa-mir-6502-5p
SAMD9L pp hsa-mir-6508-5p
SAMD9L pp hsa-mir-1301-5p
SAMD9L pp hsa-mir-1343-5p
SAMD9L pp hsa-mir-6731-5p
SAMD9L pp hsa-mir-6763-5p
SAMD9L pp hsa-mir-6796-3p
SAMD9L pp hsa-mir-6810-5p
SAMD9L pp hsa-mir-6825-5p
SAMD9L pp hsa-mir-6857-5p
SAMD9L pp hsa-mir-6864-3p
SAMD9L pp hsa-mir-6867-5p
SAMD9L pp hsa-mir-8067
SAMD9L pp hsa-mir-8085
SAMD9L pp hsa-mir-450a-2-3p
PARP9 pp hsa-mir-21-5p
PARP9 pp hsa-mir-124-3p
TRIM22 pp hsa-mir-26b-5p
TRIM22 pp hsa-mir-146a-5p
TRIM22 pp hsa-mir-335-5p
IFI44 pp hsa-mir-26b-5p

IFI44 pp hsa-mir-1-3p
IFI44 pp hsa-mir-146a-5p
GMNN pp hsa-mir-26b-5p
GMNN pp hsa-mir-212-3p
GMNN pp hsa-mir-132-3p
GMNN pp hsa-mir-324-5p
GMNN pp hsa-mir-449a
GMNN pp hsa-mir-193b-3p
GMNN pp hsa-mir-449b-5p
GMNN pp hsa-mir-421
GMNN pp hsa-mir-545-5p
GMNN pp hsa-mir-513c-5p
GMNN pp hsa-mir-3133
GMNN pp hsa-mir-514b-5p
GMNN pp hsa-mir-4282
GMNN pp hsa-mir-3681-5p
GMNN pp hsa-mir-3689d
GMNN pp hsa-mir-4511
GMNN pp hsa-mir-4709-5p
GMNN pp hsa-mir-5571-5p
GMNN pp hsa-mir-6849-5p
GMNN pp hsa-mir-6851-5p
GMNN pp hsa-mir-7847-3p
DDX60 pp hsa-mir-26b-5p
DDX60 pp hsa-mir-1-3p
IFIH1 pp hsa-mir-26b-5p
IFIH1 pp hsa-mir-29b-3p
IFIH1 pp hsa-mir-15b-5p
IFIH1 pp hsa-mir-424-5p
RSAD2 pp hsa-mir-26b-5p
RSAD2 pp hsa-mir-146a-5p
B4GALT5 pp hsa-mir-30a-5p
B4GALT5 pp hsa-mir-29b-3p
B4GALT5 pp hsa-mir-30c-5p
B4GALT5 pp hsa-mir-30d-5p
B4GALT5 pp hsa-mir-34a-5p
B4GALT5 pp hsa-mir-205-5p
B4GALT5 pp hsa-mir-30b-5p
B4GALT5 pp hsa-mir-124-3p
B4GALT5 pp hsa-mir-152-3p
B4GALT5 pp hsa-mir-185-5p
B4GALT5 pp hsa-mir-299-3p
B4GALT5 pp hsa-mir-30e-5p
B4GALT5 pp hsa-mir-335-5p
B4GALT5 pp hsa-mir-329-3p
B4GALT5 pp hsa-mir-491-5p
B4GALT5 pp hsa-mir-503-5p
B4GALT5 pp hsa-mir-603
B4GALT5 pp hsa-mir-641
B4GALT5 pp hsa-mir-139-3p
B4GALT5 pp hsa-mir-362-3p
B4GALT5 pp hsa-mir-502-3p
B4GALT5 pp hsa-mir-551b-5p
B4GALT5 pp hsa-mir-939-5p

B4GALT5 pp hsa-mir-1265
B4GALT5 pp hsa-mir-2278
B4GALT5 pp hsa-mir-3150a-3p
B4GALT5 pp hsa-mir-3151-5p
B4GALT5 pp hsa-mir-3175
B4GALT5 pp hsa-mir-4306
B4GALT5 pp hsa-mir-3617-5p
B4GALT5 pp hsa-mir-3941
B4GALT5 pp hsa-mir-4447
B4GALT5 pp hsa-mir-4472
B4GALT5 pp hsa-mir-4495
B4GALT5 pp hsa-mir-4644
B4GALT5 pp hsa-mir-4764-5p
B4GALT5 pp hsa-mir-4789-3p
B4GALT5 pp hsa-mir-1273f
B4GALT5 pp hsa-mir-5195-5p
B4GALT5 pp hsa-mir-4524b-3p
B4GALT5 pp hsa-mir-561-5p
B4GALT5 pp hsa-mir-873-3p
B4GALT5 pp hsa-mir-1343-5p
B4GALT5 pp hsa-mir-6731-5p
B4GALT5 pp hsa-mir-6742-5p
B4GALT5 pp hsa-mir-6763-5p
B4GALT5 pp hsa-mir-6796-5p
B4GALT5 pp hsa-mir-6825-5p
B4GALT5 pp hsa-mir-6830-5p
B4GALT5 pp hsa-mir-6881-5p
B4GALT5 pp hsa-mir-6888-5p
B4GALT5 pp hsa-mir-7844-5p
B4GALT5 pp hsa-mir-8085
B4GALT5 pp hsa-mir-8485
SAMD9 pp hsa-mir-30a-5p
RTP4 pp hsa-mir-30a-3p
RTP4 pp hsa-mir-30e-3p
RTP4 pp hsa-mir-330-3p
RTP4 pp hsa-mir-452-5p
RTP4 pp hsa-mir-485-3p
RTP4 pp hsa-mir-30d-3p
RTP4 pp hsa-mir-4257
RTP4 pp hsa-mir-676-5p
RTP4 pp hsa-mir-3942-3p
RTP4 pp hsa-mir-4676-3p
RTP4 pp hsa-mir-4714-3p
RTP4 pp hsa-mir-539-3p
RTP4 pp hsa-mir-892c-3p
RTP4 pp hsa-mir-6835-3p
RTP4 pp hsa-mir-6847-5p
RTP4 pp hsa-mir-1-5p
SAP30 pp hsa-mir-92a-3p
SAP30 pp hsa-mir-192-5p
SAP30 pp hsa-mir-212-3p
SAP30 pp hsa-mir-215-5p
SAP30 pp hsa-mir-382-5p
SAP30 pp hsa-mir-125b-2-3p

SAP30 pp hsa-mir-335-3p
SAP30 pp hsa-mir-513b-5p
SAP30 pp hsa-mir-4457
SAP30 pp hsa-mir-4773
SAP30 pp hsa-mir-5096
HNRNPLL pp hsa-mir-92a-3p
GBP4 pp hsa-mir-92a-3p
GBP4 pp hsa-mir-378a-5p
GBP4 pp hsa-mir-340-3p
GBP4 pp hsa-mir-335-5p
GBP4 pp hsa-mir-558
GBP4 pp hsa-mir-640
GBP4 pp hsa-mir-122-3p
GBP4 pp hsa-mir-543
GBP4 pp hsa-mir-942-5p
GBP4 pp hsa-mir-1234-3p
GBP4 pp hsa-mir-1236-3p
GBP4 pp hsa-mir-1307-3p
GBP4 pp hsa-mir-1976
GBP4 pp hsa-mir-3143
GBP4 pp hsa-mir-4279
GBP4 pp hsa-mir-4633-3p
GBP4 pp hsa-mir-4638-5p
GBP4 pp hsa-mir-4722-3p
GBP4 pp hsa-mir-4752
GBP4 pp hsa-mir-6500-5p
GBP4 pp hsa-mir-6515-3p
GBP4 pp hsa-mir-6727-3p
GBP4 pp hsa-mir-6747-3p
GBP4 pp hsa-mir-6758-3p
GBP4 pp hsa-mir-6778-3p
GBP4 pp hsa-mir-6790-3p
GBP4 pp hsa-mir-6821-3p
GBP4 pp hsa-mir-6827-3p
GBP4 pp hsa-mir-6833-5p
GBP4 pp hsa-mir-6874-5p
GBP4 pp hsa-mir-7107-5p
GBP4 pp hsa-mir-7847-3p
GBP4 pp hsa-mir-3653-5p
IFIT3 pp hsa-mir-99a-5p
IFIT3 pp hsa-mir-100-5p
IFIT3 pp hsa-mir-1-3p
IFIT3 pp hsa-mir-124-3p
IFIT3 pp hsa-mir-146a-5p
IFIT3 pp hsa-mir-99b-5p
IFIT3 pp hsa-mir-337-3p
IFIT3 pp hsa-mir-202-5p
IFIT3 pp hsa-mir-512-3p
IFIT3 pp hsa-mir-92b-3p
IFIT3 pp hsa-mir-570-3p
IFIT3 pp hsa-mir-4252
IFIT3 pp hsa-mir-3613-3p
IFIT3 pp hsa-mir-3926
IFIT3 pp hsa-mir-4438

IFIT3 pp hsa-mir-4731-5p
IFIT3 pp hsa-mir-5095
IFIT3 pp hsa-mir-5003-5p
IFIT3 pp hsa-mir-5089-5p
IFIT3 pp hsa-mir-5589-5p
IFIT3 pp hsa-mir-1273g-3p
IFIT3 pp hsa-mir-3927-5p
IFIT3 pp hsa-mir-6504-3p
IFIT3 pp hsa-mir-6506-5p
IFIT3 pp hsa-mir-619-5p
IFIT3 pp hsa-mir-6807-5p
IFIT3 pp hsa-mir-7151-3p
RNF213 pp hsa-mir-101-3p
RNF213 pp hsa-mir-10a-5p
RNF213 pp hsa-mir-1-3p
RNF213 pp hsa-mir-99b-5p
RNF213 pp hsa-mir-335-5p
RNF213 pp hsa-mir-423-3p
RNF213 pp hsa-mir-582-5p
RNF213 pp hsa-mir-603
RNF213 pp hsa-mir-183-3p
RNF213 pp hsa-mir-127-5p
RNF213 pp hsa-mir-1303
RNF213 pp hsa-mir-4452
RNF213 pp hsa-mir-4496
RNF213 pp hsa-mir-4635
RNF213 pp hsa-mir-4738-3p
RNF213 pp hsa-mir-216b-3p
RNF213 pp hsa-mir-3928-5p
RNF213 pp hsa-mir-6758-5p
RNF213 pp hsa-mir-6806-3p
RNF213 pp hsa-mir-6843-3p
RNF213 pp hsa-mir-6848-3p
RNF213 pp hsa-mir-6856-5p
RNF213 pp hsa-mir-8485
GALM pp hsa-mir-129-5p
GALM pp hsa-mir-370-3p
GALM pp hsa-mir-375
GALM pp hsa-mir-498
GALM pp hsa-mir-607
GALM pp hsa-mir-335-3p
GALM pp hsa-mir-455-3p
GALM pp hsa-mir-1305
GALM pp hsa-mir-1915-3p
GALM pp hsa-mir-1976
GALM pp hsa-mir-3157-5p
GALM pp hsa-mir-4279
GALM pp hsa-mir-4444
GALM pp hsa-mir-3977
GALM pp hsa-mir-4698
GALM pp hsa-mir-4726-3p
GALM pp hsa-mir-4803
GALM pp hsa-mir-5003-3p
GALM pp hsa-mir-1306-5p

GALM pp hsa-mir-6764-5p
GALM pp hsa-mir-6840-3p
GALM pp hsa-mir-6893-3p
GALM pp hsa-mir-8055
GALM pp hsa-mir-8063
GALM pp hsa-mir-1-5p
GALM pp hsa-mir-3653-5p
OAS2 pp hsa-mir-7-5p
OAS2 pp hsa-mir-132-3p
OAS2 pp hsa-mir-185-5p
OAS2 pp hsa-mir-335-5p
OAS2 pp hsa-mir-573
OAS2 pp hsa-mir-620
OAS2 pp hsa-mir-148a-5p
OAS2 pp hsa-mir-1270
OAS2 pp hsa-mir-2114-3p
OAS2 pp hsa-mir-4306
OAS2 pp hsa-mir-3616-5p
OAS2 pp hsa-mir-4531
OAS2 pp hsa-mir-4635
OAS2 pp hsa-mir-4644
OAS2 pp hsa-mir-4670-3p
OAS2 pp hsa-mir-5192
OAS2 pp hsa-mir-6751-3p
OAS2 pp hsa-mir-6823-3p
OAS2 pp hsa-mir-1199-5p
DDX58 pp hsa-mir-10b-5p
DDX58 pp hsa-mir-218-5p
STAT1 pp hsa-mir-34a-5p
STAT1 pp hsa-mir-203a-3p
STAT1 pp hsa-mir-223-3p
STAT1 pp hsa-mir-140-5p
STAT1 pp hsa-mir-145-5p
STAT1 pp hsa-mir-146a-5p
STAT1 pp hsa-mir-150-5p
STAT1 pp hsa-mir-155-5p
STAT1 pp hsa-mir-450a-5p
STAT1 pp hsa-mir-501-5p
STAT1 pp hsa-mir-605-5p
STAT1 pp hsa-mir-615-3p
STAT1 pp hsa-mir-653-5p
STAT1 pp hsa-mir-500a-5p
STAT1 pp hsa-mir-1183
STAT1 pp hsa-mir-4693-5p
STAT1 pp hsa-mir-203b-3p
STAT1 pp hsa-mir-5009-3p
STAT1 pp hsa-mir-7158-3p
RNF169 pp hsa-mir-34a-5p
NCOA7 pp hsa-mir-181a-5p
NCOA7 pp hsa-mir-181b-5p
NCOA7 pp hsa-mir-181c-5p
NCOA7 pp hsa-mir-375
NCOA7 pp hsa-mir-181d-5p
NCOA7 pp hsa-mir-498

NCOA7 pp hsa-mir-516b-3p
NCOA7 pp hsa-let-7a-3p
NCOA7 pp hsa-let-7b-3p
NCOA7 pp hsa-let-7f-1-3p
NCOA7 pp hsa-let-7f-2-3p
NCOA7 pp hsa-mir-32-3p
NCOA7 pp hsa-mir-543
NCOA7 pp hsa-mir-516a-3p
NCOA7 pp hsa-mir-1976
NCOA7 pp hsa-mir-2115-5p
NCOA7 pp hsa-mir-4262
NCOA7 pp hsa-mir-4279
NCOA7 pp hsa-mir-4722-3p
NCOA7 pp hsa-mir-4789-5p
NCOA7 pp hsa-mir-5571-5p
NCOA7 pp hsa-mir-5680
NCOA7 pp hsa-mir-1185-2-3p
NCOA7 pp hsa-mir-1185-1-3p
NCOA7 pp hsa-mir-98-3p
NCOA7 pp hsa-mir-6727-3p
NCOA7 pp hsa-mir-6728-3p
NCOA7 pp hsa-mir-6747-3p
NCOA7 pp hsa-mir-6778-3p
NCOA7 pp hsa-mir-7162-5p
NCOA7 pp hsa-mir-3653-5p
IFIT1 pp hsa-mir-203a-3p
IFIT1 pp hsa-mir-1-3p
IFIT1 pp hsa-mir-126-5p
IFIT1 pp hsa-mir-146a-5p
IFIT1 pp hsa-mir-375
IFIT1 pp hsa-mir-380-3p
IFIT1 pp hsa-mir-526b-5p
IFIT1 pp hsa-mir-578
IFIT1 pp hsa-mir-335-3p
IFIT1 pp hsa-mir-1237-3p
IFIT1 pp hsa-mir-1248
IFIT1 pp hsa-mir-2054
IFIT1 pp hsa-mir-4795-3p
IFIT1 pp hsa-mir-1250-3p
IFIT1 pp hsa-mir-6868-3p
IFIT1 pp hsa-mir-6516-3p
IFIT2 pp hsa-mir-1-3p
IFIT2 pp hsa-mir-124-3p
IFIT2 pp hsa-mir-645
IFIT2 pp hsa-mir-650
IFIT2 pp hsa-mir-193b-5p
OASL pp hsa-mir-1-3p
OASL pp hsa-mir-124-3p
OASL pp hsa-mir-146a-5p
HERC5 pp hsa-mir-1-3p
HERC5 pp hsa-mir-3529-3p
EIF2AK2 pp hsa-mir-15b-5p
EIF2AK2 pp hsa-mir-124-3p
EIF2AK2 pp hsa-mir-375

EIF2AK2 pp hsa-mir-485-5p
EIF2AK2 pp hsa-mir-202-5p
EIF2AK2 pp hsa-mir-510-5p
EIF2AK2 pp hsa-mir-34b-3p
EIF2AK2 pp hsa-mir-513a-3p
EIF2AK2 pp hsa-mir-574-5p
EIF2AK2 pp hsa-mir-298
EIF2AK2 pp hsa-mir-877-5p
EIF2AK2 pp hsa-mir-665
EIF2AK2 pp hsa-mir-940
EIF2AK2 pp hsa-mir-4282
EIF2AK2 pp hsa-mir-3614-5p
EIF2AK2 pp hsa-mir-3646
EIF2AK2 pp hsa-mir-3929
EIF2AK2 pp hsa-mir-4433a-3p
EIF2AK2 pp hsa-mir-4459
EIF2AK2 pp hsa-mir-4478
EIF2AK2 pp hsa-mir-4419b
EIF2AK2 pp hsa-mir-3127-3p
EIF2AK2 pp hsa-mir-3664-3p
EIF2AK2 pp hsa-mir-4649-3p
EIF2AK2 pp hsa-mir-4652-3p
EIF2AK2 pp hsa-mir-4695-5p
EIF2AK2 pp hsa-mir-4698
EIF2AK2 pp hsa-mir-4722-5p
EIF2AK2 pp hsa-mir-4762-5p
EIF2AK2 pp hsa-mir-4768-3p
EIF2AK2 pp hsa-mir-4769-3p
EIF2AK2 pp hsa-mir-5196-3p
EIF2AK2 pp hsa-mir-5586-5p
EIF2AK2 pp hsa-mir-513c-3p
EIF2AK2 pp hsa-mir-495-5p
EIF2AK2 pp hsa-mir-3606-3p
EIF2AK2 pp hsa-mir-4743-3p
EIF2AK2 pp hsa-mir-6500-3p
EIF2AK2 pp hsa-mir-1252-3p
EIF2AK2 pp hsa-mir-6756-3p
EIF2AK2 pp hsa-mir-6808-5p
EIF2AK2 pp hsa-mir-6832-3p
EIF2AK2 pp hsa-mir-6867-5p
EIF2AK2 pp hsa-mir-6884-5p
EIF2AK2 pp hsa-mir-6893-5p
EIF2AK2 pp hsa-mir-7158-5p
EIF2AK2 pp hsa-mir-7160-5p
EIF2AK2 pp hsa-mir-8063
BATF2 pp hsa-mir-122-5p
BATF2 pp hsa-mir-375
BATF2 pp hsa-mir-335-5p
GBP1 pp hsa-mir-124-3p
IFI44L pp hsa-mir-124-3p
IFI44L pp hsa-mir-146a-5p
IFI44L pp hsa-mir-15b-3p
IFI44L pp hsa-mir-146a-3p
IFI44L pp hsa-mir-34b-3p

IFI44L pp hsa-mir-1248
IFI44L pp hsa-mir-4256
IFI44L pp hsa-mir-3921
IFI44L pp hsa-mir-4653-5p
IFI44L pp hsa-mir-4768-5p
IFI44L pp hsa-mir-301a-5p
IFI44L pp hsa-mir-4750-3p
IFI44L pp hsa-mir-6759-3p
IFI44L pp hsa-mir-6809-3p
IFI44L pp hsa-mir-6817-3p
IFI44L pp hsa-mir-6833-3p
IFI44L pp hsa-mir-6873-3p
IFI44L pp hsa-mir-7110-3p
IFI44L pp hsa-mir-301b-5p
PARP14 pp hsa-mir-124-3p
FRMD3 pp hsa-mir-124-3p
FRMD3 pp hsa-mir-377-3p
FRMD3 pp hsa-mir-342-3p
FRMD3 pp hsa-mir-1279
FRMD3 pp hsa-mir-3613-3p
FRMD3 pp hsa-mir-4768-5p
FRMD3 pp hsa-mir-5007-3p
FRMD3 pp hsa-mir-6817-3p
FRMD3 pp hsa-mir-6833-3p
FRMD3 pp hsa-mir-6873-3p
FRMD3 pp hsa-mir-7110-3p
FRMD3 pp hsa-mir-8064
FRMD3 pp hsa-mir-8485
OAS3 pp hsa-mir-143-3p
OAS3 pp hsa-mir-502-5p
OAS3 pp hsa-mir-4708-5p
OAS3 pp hsa-mir-4770
OAS3 pp hsa-mir-1273f
OAS3 pp hsa-mir-6088
IRAK2 pp hsa-mir-146a-5p
IRAK2 pp hsa-mir-335-5p
IRAK2 pp hsa-mir-485-5p
IRAK2 pp hsa-mir-512-5p
IRAK2 pp hsa-mir-520g-3p
IRAK2 pp hsa-mir-520h
IRAK2 pp hsa-mir-510-5p
IRAK2 pp hsa-mir-661
IRAK2 pp hsa-mir-766-3p
IRAK2 pp hsa-mir-34b-3p
IRAK2 pp hsa-mir-508-5p
IRAK2 pp hsa-mir-665
IRAK2 pp hsa-mir-940
IRAK2 pp hsa-mir-1262
IRAK2 pp hsa-mir-1827
IRAK2 pp hsa-mir-4324
IRAK2 pp hsa-mir-3609
IRAK2 pp hsa-mir-3614-5p
IRAK2 pp hsa-mir-3664-5p
IRAK2 pp hsa-mir-3672

IRAK2 pp hsa-mir-3929
IRAK2 pp hsa-mir-4433a-3p
IRAK2 pp hsa-mir-548ah-5p
IRAK2 pp hsa-mir-4459
IRAK2 pp hsa-mir-4478
IRAK2 pp hsa-mir-4419b
IRAK2 pp hsa-mir-4504
IRAK2 pp hsa-mir-4522
IRAK2 pp hsa-mir-3664-3p
IRAK2 pp hsa-mir-4649-3p
IRAK2 pp hsa-mir-4695-5p
IRAK2 pp hsa-mir-4701-3p
IRAK2 pp hsa-mir-4722-5p
IRAK2 pp hsa-mir-4768-3p
IRAK2 pp hsa-mir-873-3p
IRAK2 pp hsa-mir-6500-3p
IRAK2 pp hsa-mir-6510-3p
IRAK2 pp hsa-mir-6511a-5p
IRAK2 pp hsa-mir-6512-3p
IRAK2 pp hsa-mir-1910-3p
IRAK2 pp hsa-mir-6720-5p
IRAK2 pp hsa-mir-6736-5p
IRAK2 pp hsa-mir-6742-3p
IRAK2 pp hsa-mir-6761-5p
IRAK2 pp hsa-mir-6776-5p
IRAK2 pp hsa-mir-6808-5p
IRAK2 pp hsa-mir-6864-3p
IRAK2 pp hsa-mir-6884-5p
IRAK2 pp hsa-mir-6893-5p
IRAK2 pp hsa-mir-7160-5p
EPSTI1 pp hsa-mir-146a-5p
EPSTI1 pp hsa-mir-654-5p
IFI6 pp hsa-mir-375
IFI6 pp hsa-mir-1225-3p
OAS1 pp hsa-mir-335-5p
XAF1 pp hsa-mir-335-5p
VNN1 pp hsa-mir-526b-5p
VNN1 pp hsa-mir-924
VNN1 pp hsa-mir-1224-5p
VNN1 pp hsa-mir-1289
VNN1 pp hsa-mir-3198
VNN1 pp hsa-mir-4294
VNN1 pp hsa-mir-4309
VNN1 pp hsa-mir-3915
VNN1 pp hsa-mir-4689
VNN1 pp hsa-mir-4751
VNN1 pp hsa-mir-4796-5p
VNN1 pp hsa-mir-5004-5p
VNN1 pp hsa-mir-6858-5p
LY6E pp hsa-mir-608
LY6E pp hsa-mir-654-5p
LY6E pp hsa-mir-486-3p
LY6E pp hsa-mir-541-3p
LY6E pp hsa-mir-744-5p

LY6E pp hsa-mir-877-3p
LY6E pp hsa-mir-1909-3p
LY6E pp hsa-mir-2861
LY6E pp hsa-mir-3665
LY6E pp hsa-mir-3689d
LY6E pp hsa-mir-4651
LY6E pp hsa-mir-4736
LY6E pp hsa-mir-6132
LY6E pp hsa-mir-6721-5p
LY6E pp hsa-mir-6722-3p
LY6E pp hsa-mir-6732-5p
LY6E pp hsa-mir-6752-5p
LY6E pp hsa-mir-6756-5p
LY6E pp hsa-mir-6766-5p
LY6E pp hsa-mir-6798-5p
LY6E pp hsa-mir-6836-5p
LY6E pp hsa-mir-6842-5p
LY6E pp hsa-mir-6851-5p
LY6E pp hsa-mir-6880-5p
LY6E pp hsa-mir-7110-5p
LY6E pp hsa-mir-7847-3p
