# Supplementary figures and images for: Exploring the shared pathogenic mechanisms of tuberculosis and COVID-19: emphasizing the role of VNN1 in severe COVID-19
Source: Front Cell Infect Microbiol. 2024 Nov 21;14:1453466. doi: 10.3389/fcimb.2024.1453466 (PMC11618882; doi:10.3389/fcimb.2024.1453466)

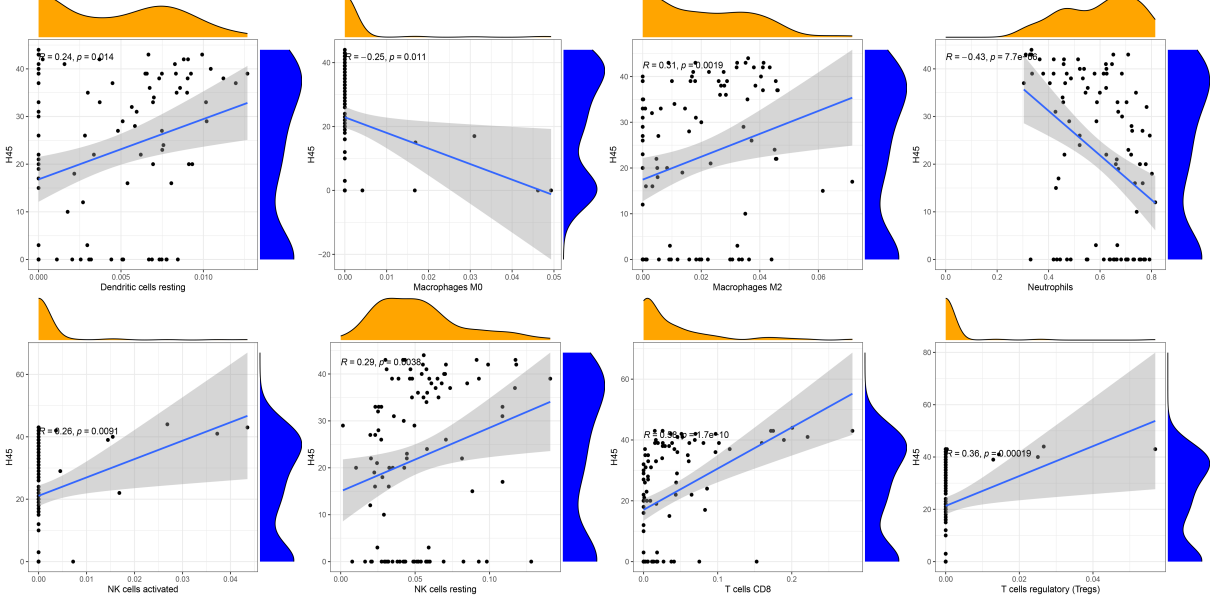

Supplement: Supplementary file 7 [file DataSheet7.pdf]

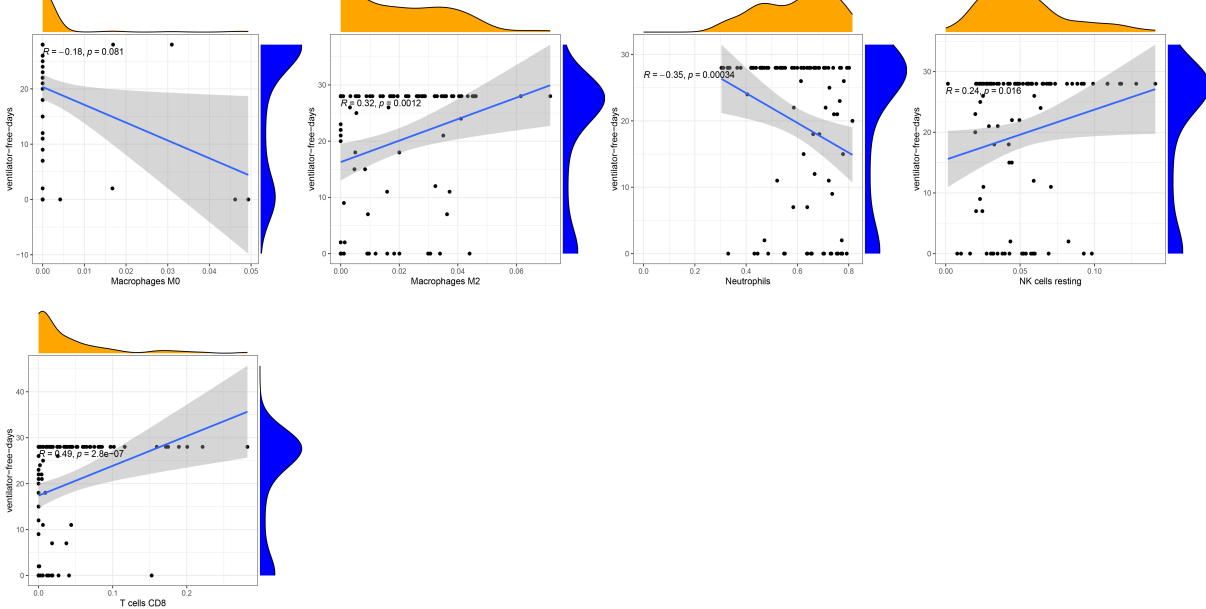

Supplement: Supplementary file 8 [file DataSheet8.pdf]
