# Supplementary material for: Exploring the shared pathogenic mechanisms of tuberculosis and COVID-19: emphasizing the role of VNN1 in severe COVID-19
Source: Front Cell Infect Microbiol. 2024 Nov 21;14:1453466. doi: 10.3389/fcimb.2024.1453466 (PMC11618882; doi:10.3389/fcimb.2024.1453466)
Supplement: Supplementary file 9 [file DataSheet9.pdf]

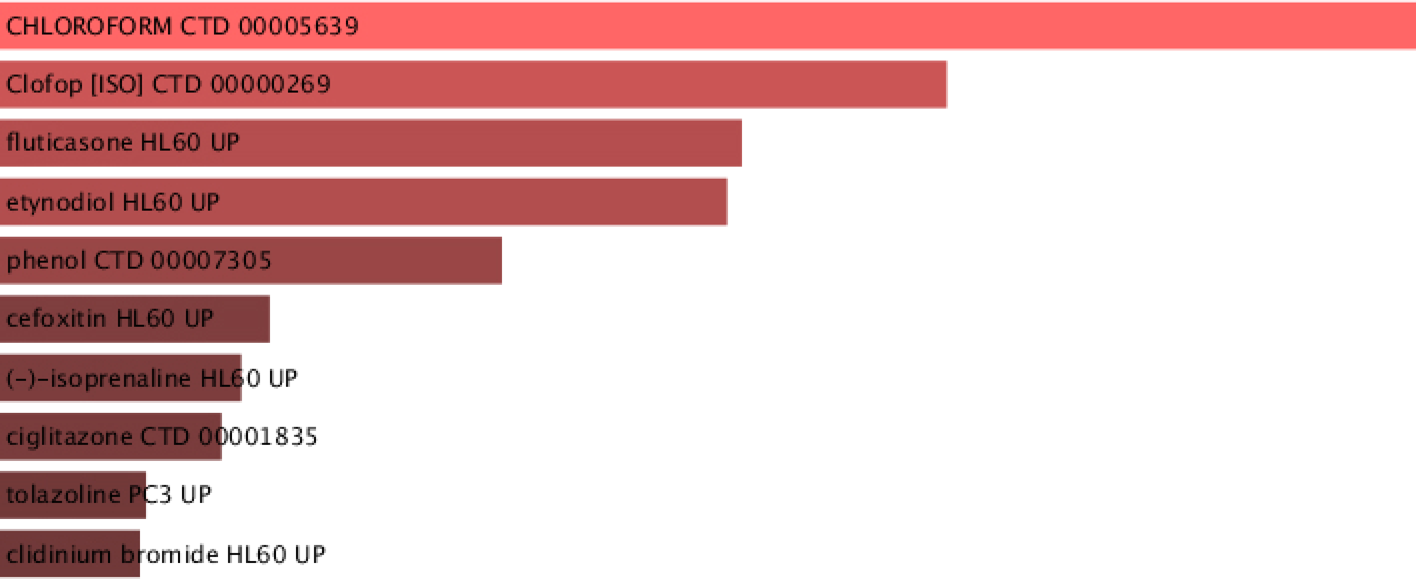
CHLOROFORM CTD 00005639
Clofop [ISO] CTD 00000269
fluticasone HL60 UP
etynodiol HL60 UP
phenol CTD 00007305
cefoxitin HL60 UP
(-)-isoprenaline HL60 UP
ciglitazone CTD 00001835
tolazoline PC3 UP
clidinium bromide HL60 UP
